# From Formation to Failure: The Role of Hydrogen Peroxide in Proton Exchange Membrane Technologies

**DOI:** 10.1021/acscatal.5c08411

**Published:** 2026-02-16

**Authors:** Tingting Mo, Christopher M. Zalitis, Colleen Jackson, Enrico Petrucco, Jonathan Sharman, Anthony R. J. Kucernak

**Affiliations:** † Imperial College London, Department of Chemistry, Molecular Science Research Hub, London W12 0BZ, U.K.; ‡ 263220 Johnson Matthey Technology Centre, Blounts Court Road, Sonning Common, Reading RG4 9NH, U.K.

**Keywords:** proton exchange membrane fuel cells, proton exchange membrane water electrolyzers, gas crossover, H_2_O_2_, degradation

## Abstract

Hydrogen peroxide is a catalytic byproduct in proton exchange membrane fuel cells (PEMFCs) and proton exchange membrane water electrolyzers (PEMWEs). It may be produced as a side product of the electrochemical processes occurring at the cathode in PEMFCs, or at the anode in PEMWEs, or it may be produced due to gas crossover through either catalytic chemical or electrocatalytic processes. The challenge posed by H_2_O_2_ is its catalytic decomposition into highly reactive hydroxyl and peroxyl radicals, which trigger cascading degradation of critical components. This degradation directly compromises device efficiency and shortens the lifespan, representing a limiting factor in the durability of PEMFCs and PEMWEs. However, existing methods for detecting and quantifying in situ H_2_O_2_ generation are limited in their ability to accurately reflect real operating conditions (e.g., high current densities, mixed reactant environments), hindering a complete understanding of its dynamic (electro)­catalytic formation and impact. To address these gaps and advance the performance of hydrogen-based energy technologies, a comprehensive analysis of H_2_O_2_ (electro)­catalytic generation mechanisms, detrimental effects, and catalytic mitigation strategies is essential. In this work, we systematically review recent progress in H_2_O_2_ research for PEMFCs and PEMWEs, focusing on (1) underlying H_2_O_2_ (electro)­catalytic formation mechanisms, (2) the role of gas crossover in (electro)­catalytic H_2_O_2_ formation, (3) current detection techniques (and their limitations), and (4) emerging catalytic strategies for suppressing damage due to H_2_O_2_. This review highlights the need for improved in situ detection tools and targeted suppression approaches to enhance the reliability and longevity of PEMFCs and PEMWEs—important technologies for hydrogen-based energy systems.

## Graphical abstract

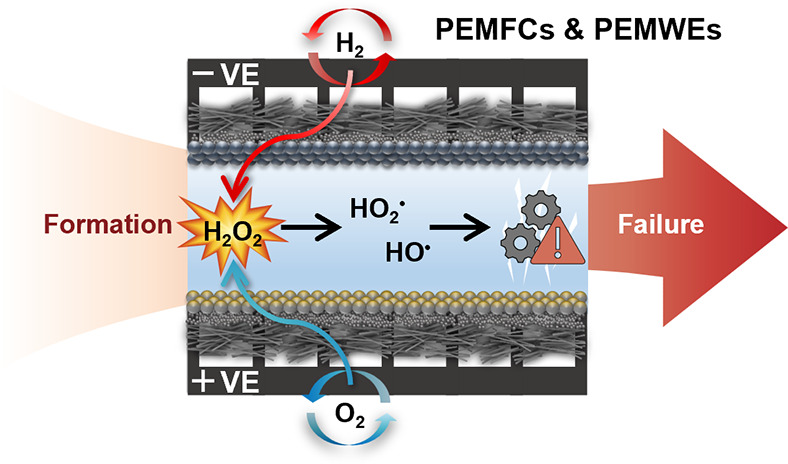

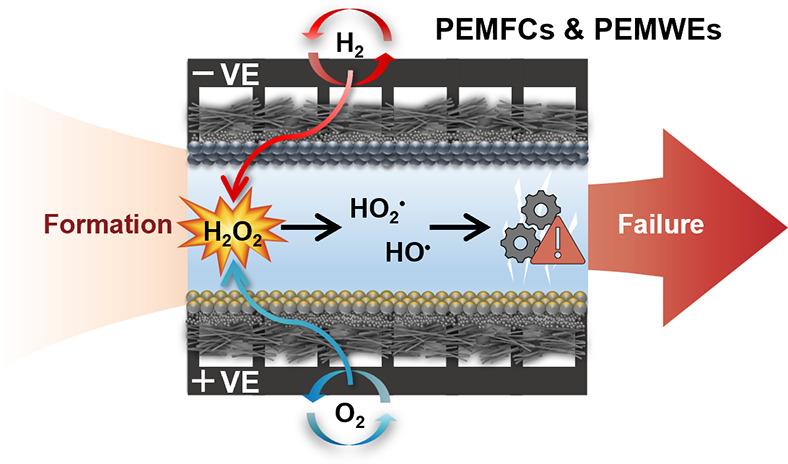

## 1 Introduction

To curb global warming and achieve net-zero emissions by 2050, many countries are accelerating the transition from fossil fuels to renewable energy, prompting growing interest in hydrogen as a long-term, energy-dense, zero-carbon-at-use energy carrier.^1–3^ Various types of fuel cells and electrolyzers have been developed which can be categorized based on the type of ionic species used (OH^–^, O^2–^, and H^+^), leading to three major types of technologies: alkaline, solid oxide, and proton exchange membrane (PEM). In this review, we consider only the low-temperature systems and, most specifically, the polymer electrolyte systems. PEM-based technologies evolved from liquid phosphoric acid and liquid alkaline (KOH) versions due to the development of solid polymer electrolytes. The key technical features of PEMFCs and PEMWEs are outlined in Table 1. The polymer electrolytes achieved a significant boost in stability due to the introduction of a stable perfluorosulfonic cation exchange electrolyte which had been developed for the Chlor-Alkali industry, although there remain challenging targets for low stack degradation rates by 2030 of 0.2 and 0.12%/kh (kh = 1000 hours) for PEMFCs and PEMWEs, respectively, Table 1. As we will see below, hydrogen peroxide generation and subsequent formation of reactive oxygen species (ROS) are among the major issues which contribute to stack degradation.

**Table 1:** Comparison of MEA Composition, Operation Conditions, and Degradations between PEMFCs and PEMWEs^4,5^

	**PEMFCs**	**PEMWEs**
Electrode Reactions	Cathode: O_2(g)_ + 4H_(aq)_^+^ + 4e^–^ → 2H_2_O_(l)_ (ORR)	Cathode: 2H_(aq)_^+^ + 2e^–^ → H_2(g)_ (HER)
	Anode: H_2(g)_ → 2H_(aq)_^+^ + 2e^–^ (HOR)	Anode: 2H_2_O_(l)_ → O_2(g)_ + 4H_(aq)_^+^ + 4e^–^ (OER)
Catalyst Requirements^4^	Cathode: Pt (0.1–0.4 mg_Pt_ cm^–2^)	Cathode: Pt/C (0.5–1 mg_Pt_ cm^–2^)
	Anode: Pt (0.05–0.1 mg_Pt_ cm^–2^)	Anode: IrO_*x*_ (1.0–4.0 mg_Ir_ cm^–2^)
Membrane Thickness	10–50 μm	100–180 μm
Porous Transport Layer^5^	Cathode: Carbon Paper	Cathode: Carbon Paper
	Anode: Carbon Paper	Anode: Ti Fiber or Sinter
Operating Current Density^4^	1.5 A cm^–2^,	2.2 A cm^–2^,
	>2.3 A cm^–2^ (2030)	3.0 A cm^–2^ (2030)
Operating Potential^4^	0.65 V	1.7 V
Operating Temperature^5^	80 °C	60–80 °C
Gas Conditions^5^	Cathode: O_2_ (Air)	Cathode: H_2_
	Anode: H_2_	Anode: O_2_, Water (asymmetric pressure)
Stack Degradation Rate (%/1000 h)^4^	0.4 (SoA), 0.2 (2030)	0.19 (SoA), 0.12 (2030)

In PEMFCs, hydrogen is used as fuel, supplied to the anode, and split into protons and electrons through the hydrogen oxidation reaction (HOR), where the protons travel through the proton-conductive membranes. The protons then react with oxygen and electrons at the cathode to produce water via the oxygen reduction reaction (ORR). Electro-osmotic drag transports water from the anode to the cathode and is countered by water back-diffusion in the opposite direction. These processes are important in keeping the membrane hydrated to facilitate proton conduction and to prevent the cathode from flooding and the anode from drying out. A schematic illustration of a PEMFC is shown in Figure 1, left.

**Figure 1:**
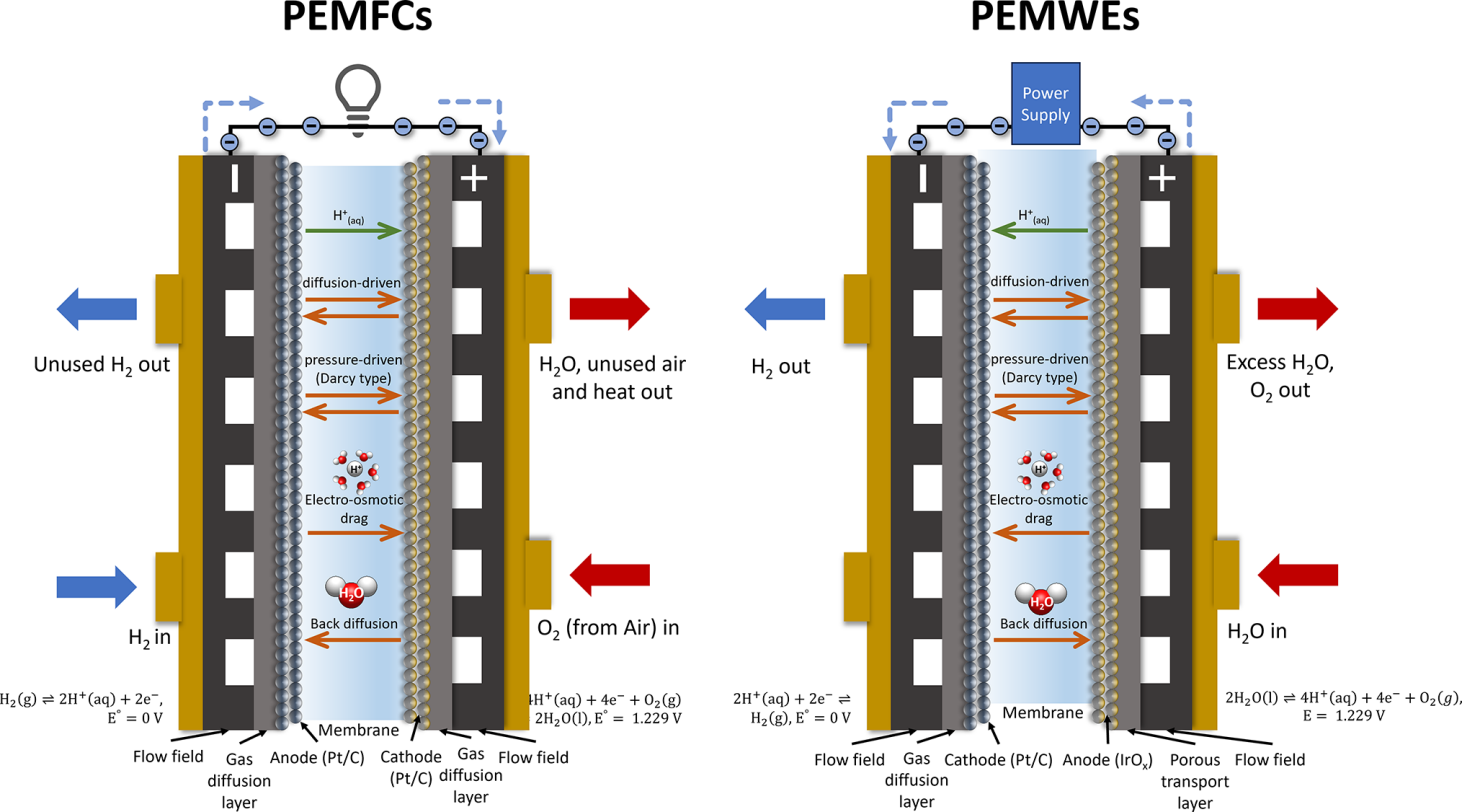
Schematic illustration of cross section of a single cell of (a) a PEM fuel cell and (b) a PEM water electrolyzer showing the important transport processes.

Green hydrogen is produced by electrolysis in which water is split into oxygen and hydrogen using renewable energy sources. It offers an approach to produce valuable feedstock and capture and store excess renewable energy, providing a high level of flexibility with fast startup and shutdown. PEMWEs offer high current densities, increased rate of hydrogen production, high gas purity (up to 99.99%), and provision at pressure, high energy efficiency (50–80 kWh kg^–1^), and extended lifetime (50,000–80,000 h), all under realistic operating conditions.^6^ In PEMWEs, the process is the reverse of that occurring in PEMFCs: water is supplied to the anode and split into oxygen, protons, and electrons through the oxygen evolution reaction (OER). Protons are transported through the membrane, while electrons are emitted from the anode through the external power circuit to provide the driving force of the reaction, which is set by the cell voltage. The recombination of electrons and protons occurs through the hydrogen evolution reaction (HER) at the cathode. A simplified schematic of the membrane electrode assembly (MEA) for commercial PEMWEs is shown in Figure 1, right. The hydrogen side is the anode for the fuel cell and the cathode for the electrolyzer, and the oxygen side is the cathode for the fuel cell and the anode for the electrolyzer. To avoid confusion, in the following sections, the terms “hydrogen electrode” and “oxygen electrode” are used instead of “cathode” and “anode”.

The MEA constitutes the central component of both PEMFCs and PEMWEs. Understanding the degradation mechanism of the MEA is crucial for enhancing its durability. Perfluorosulfonic acid (PFSA) membranes, employed as solid electrolytes within the MEA, allow proton transportation and exhibit a limited degree of gas permeability. All gas transport processes within the membrane are summarized in Figure 1. A thinner membrane is advantageous due to its lower internal resistance, resulting in reduced operating voltage, decreased MEA costs due to a reduction in material usage, and improved back-transport and distribution of water. However, it also has certain limitations, such as increased gas crossover in both directions, greater susceptibility to damage due to a reduced amount of material, and lower mechanical robustness. Multiple factors influence the degree of crossover, including temperature, gas partial pressure, differential pressure in the electrode compartments, gas diffusion coefficient in the membrane, membrane hydration, and additives within the membrane, such as recombination catalysts. These are discussed in greater detail in Section 3.

Electrochemical production of H_2_O_2_ is a widely studied reaction, and on select catalysts (e.g., Au- and Hg- based catalysts) it often emerges as the dominant product due to their high selectivity.^14,15^ However, it is an undesired product in PEMWEs and PEMFCs. Understanding peroxide formation and the conditions that favors its formation in PEM technologies is vital, as it sits at the nexus of durability, activity, and the MEA design. Species transport through membrane, ionomer, or porous layers can occur by diffusion, pressure-driven (Darcy-type) flow, migration, and electro-osmotic drag (Figure 2, left corner). For gases under normal operating conditions, diffusion dominates, while Darcy-type flow (at least in PFSA materials) is typically a minor contribution. However, this may change if the membrane suffers from a pinhole or other defect. Electro-osmotic drag of water in the membrane will provide an extra advective flux of gas molecules, but this aspect is also minor. For water, the main pathways are electro-osmosis and diffusion, which typically act in opposition. For protons and charged metal ions (e.g., Pt^2+^, Co^2+^, Fe^2+^) in ion exchange materials, migration is usually the controlling mechanism, though diffusion can also matter in other (non-ion-exchanged) matrices. For uncharged or radical species (e.g., H_2_O_2_, reactive oxygen species (ROS)), diffusion is generally most important, with electro-osmosis potentially contributing as well, although the short lifespan of ROS means that they are unlikely to travel far from their point of generation. Production of H_2_O_2_ through both gas crossover and electrochemical processes at different points in the MEA is summarized in six different subcases (cases a–f, detailed in Section 4.1) in Figure 2. Peroxide can form via electrochemical routes, i.e., the two-electron reduction of O_2_ at the (b) hydrogen electrode or (e) oxygen electrodes or (f) the two-electron oxidation of H_2_O at the oxygen electrode, and via chemical routes driven by H_2_ or O_2_ crossover, producing peroxide at (a) the hydrogen electrode, (d) the oxygen electrode, or (c) recombination sites in the membrane.

**Figure 2:**
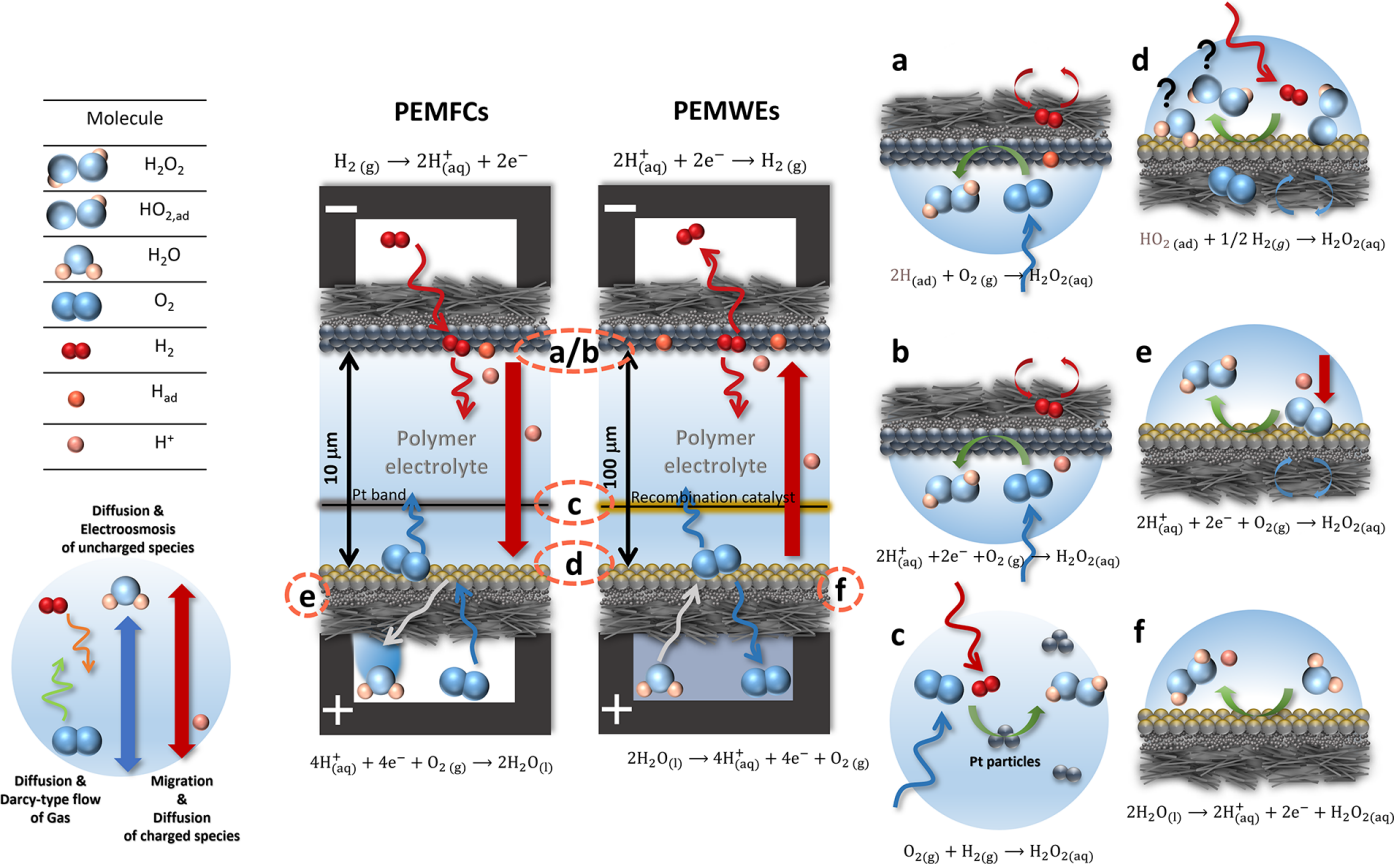
Schematic summary of the different hydrogen peroxide formation mechanisms in PEMFCs (left) and PEMWEs (right). Electrochemical processes include the two-electron reduction of oxygen on the (b) hydrogen^7,8^ or (e) oxygen electrodes^9^ or (f) the two-electron oxidation of water on the oxygen electrode.^10^ Chemical processes involve hydrogen or oxygen gas crossover and lead to formation on the (a) hydrogen electrode,^11^ (d) oxygen electrode,^11^ or (c) recombination sites in the membrane.^12,13^

Crossover of oxygen and hydrogen leads to the chemical/electrochemical formation of hydrogen peroxide; Figure 3 illustrates in cartoon form the negative feedback process occurring through gas crossover and membrane degradation processes. Oxygen may undergo one-electron reduction to superoxide and hydroperoxyl radicals; although hydroperoxyl radicals are short-lived in acidic media and disproportionate to peroxide, this nonetheless represents a direct radical pathway that should be considered alongside peroxide chemistry. A more detailed discussion is provided in Section 4.

**Figure 3:**
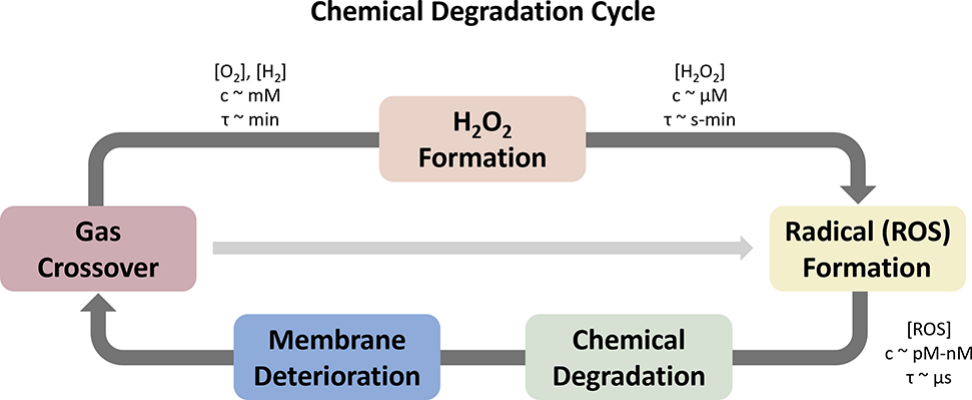
Chemical degradation cycle for fuel cells and electrolyzers, providing indicative concentrations (c) and lifetimes (τ) of the respective species, showing the major pathway (through H_2_O_2_) and minor pathway (avoiding H_2_O_2_ production). Adapted with permission from refs 17–19 . Copyright 2019 Elsevier; Copyright 2005 The Electrochemical Society; and Copyright 2011 The Electrochemical Society.

Hydrogen peroxide itself is a strong oxidizing agent which has a moderately long lifespan within the polymer environment (estimated to be minutes), but it can then undergo reductive decomposition to produce short-lived hydroxyl radicals (and, through this, hydroperoxyl radicals) in the presence of metal impurities (e.g., Fe^2+^, Cu^2+^, Ti^3+^, etc.). These impurities may originate from impurities in the catalyst or support, degradation of metal components in the stack, or external contamination. The resulting radicals are even more oxidizing than the hydrogen peroxide, although they correspondingly have much short half-lives (∼μs), and can damage the membrane, leading to its deterioration.^16^ Their short half-lives mean that they will not travel far from their point of production. Electrochemical reduction of crossover oxygen at the hydrogen electrode may produce hydroperoxyl radicals (especially in electrolyzers due to the lower potential of the cathode), and this represents a possible pathway for radical production, avoiding hydrogen peroxide. Although direct combination of hydrogen and oxygen gases seems like a potential pathway for production of hydroxyl radicals (see below), such a process has not been observed to occur. Membrane degradation leads to increased crossover, which accelerates hydrogen peroxide generation, leading to eventual failure.

This review seeks to provide deeper insights into electrochemical and chemical hydrogen peroxide formation in PEMWEs and PEMFCs. It offers a comprehensive exploration of the thermodynamics and mechanistic pathways leading to hydrogen peroxide generation, elucidating critical factors that influence gas crossover and subsequent peroxide formation and explaining how these processes ultimately lead to the failure of the MEA. Given the typically low concentration of H_2_O_2_ produced within the MEA, this review examines and evaluates current methodologies for accurately detecting and quantifying trace hydrogen peroxide. Moving beyond fundamental studies, recent advancements and strategies aimed at mitigating H_2_O_2_ formation are discussed to provide insights into preventing MEA degradation and failure. Finally, unsolved challenges and future research directions are proposed, with the aim of shedding light on strategies to enhance the lifetime of PEM devices.

## 2 Fundamentals

### 2.1 Thermodynamics of Solution Phase Electrochemistry and Processes

The thermodynamics of hydrogen peroxide and other ROS production in free solution are illustrated by Table 2, which shows the different ways in which hydrogen peroxide can be produced through both electrochemical (oxygen reduction, water oxidation, indirectly though superoxide/hydroperoxyl radical formation), with their corresponding standard potentials (*E* ^o^’s) given, and chemical (direct hydrogen/oxygen combination, reverse peroxide decomposition, hydroperoxyl disproportionation), with their associated free energies of reaction (Δ_r_ *G* ^o^’s) and the acid constants (p*K*_a_’s) of the corresponding acidic species. The practical importance of individual reactions is condition-dependent (e.g., operating potential windows, local pH/water activity, catalyst surface, and impurity content), and the most relevant pathways for PEMFC/PEMWE operation are discussed in Sections 2.2 and 2.3. The electrochemical potential for forming a surface-adsorbed intermediate can differ from that of the corresponding solvated species because the adsorption/desorption energies contribute to the overall interfacial energetics. Therefore, comparisons between solution-phase thermodynamics and surface-adsorbed pathways should be made with caution. Further details are provided in the Supporting Information (Figure S1). In Table 2, all electrochemical reactions are written as reductions, although the reverse reactions may be the ones of interest.

**Table 2:** Thermodynamics of the Production of Hydrogen Peroxide and Other Reactive Oxygen Species (ROS) in Free Solution^7,20–24^ ^a^_,_ ^a^

**Type**	**Reaction**		**Δ_r_ *G* ^o^ (kJ mol^–1^)**	
**Electrochemical – Oxygen Reduction**				***E* ^o^ (V)**
To water:	O_2(g)_ + 4H_(aq)_^+^ + 4e^–^ ⇌ 2H_2_O_(l)_	(1)	–474.3	1.229
To hydrogen peroxide:	O_2(g)_ + 2H_(aq)_^+^ + 2e^–^ ⇌ H_2_O_2(aq)_	(2)	–134.1	0.695
To hydroperoxyl radical:	O_2(g)_ + H_(aq)_^+^ + e^–^ ⇌ HO_2(aq)_^•^	(3)	7	–0.07

**Electrochemical – Water Oxidation**				***E* ^o^ (V)**
To oxygen:	*See eq 1 above*			
To hydrogen peroxide:	H_2_O_2(aq)_ + 2H_(aq)_^+^ + 2e^–^ ⇌ 2H_2_O_(l)_	(4)	–340.3	1.763
To hydroxyl radical:	HO_(aq)_^•^ + H_(aq)_^+^ + e^–^ ⇌ H_2_O_(l)_	(5)	–263	2.73

**Electrochemical – Interconversion**				***E* ^o^ (V)**
Hydroperoxyl radical reduction to peroxide:	HO_2(aq)_^•^ + H_(aq)_^+^ + e^–^ ⇌ H_2_O_2(aq)_	(6)	–141	1.46
Hydroperoxyl to hydroxyl radical:	HO_2(aq)_^•^ + 2H_(aq)_^+^ + 2e^–^ ⇌ HO_(aq)_^•^ + H_2_O_(l)_	(7)	–218	1.13
Hydroperoxyl radical to water:	HO_2(aq)_^•^ + 3H_(aq)_^+^ + 3e^–^ ⇌ 2H_2_O_(l)_	(8)	–481	1.66

**Chemical**				
Direct combination to water:	O_2(g)_ + 2H_2(aq)_ ⇌ 2H_2_O_(l)_	(9)	–474.3	
Direct combination to hydrogen peroxide:	O_2(g)_ + H_2(g)_ ⇌ H_2_O_2(aq)_	(10)	–134.1	
Direct combination to hydroxyl radical:	O_2(g)_ + H_2(g)_ ⇌ 2HO_(aq)_^•^	(11)	53	
Direct combination to hydroperoxyl and hydrogen radicals:	O_2(g)_ + H_2(g)_ ⇌ HO_2,(aq)_^•^ + H_(aq)_^•^	(12)	230	
Peroxide disproportionation:	2H_2_O_2(aq)_ ⇌ 2H_2_O_(l)_ + O_2(g)_	(13)	–206.2	
Peroxide homolysis to hydroxyl radicals:	H_2_O_2(aq)_ ⇌ 2HO_(aq)_^•^	(14)	187	
Peroxide homolysis to hydroperoxyl and hydrogen radicals:	H_2_O_2(aq)_ ⇌ HO_2,(aq)_^•^ + H_(aq)_^•^	(15)	364	
Hydroperoxyl radical disproportionation:	2HO_2(aq)_^•^ ⇌ H_2_O_2(aq)_ + O_2(g)_	(16)	–148	

**Acid Dissociation**				**p*K*_a_**
Water dissociation:	H_2_O_(l)_ ⇌ HO_(aq)_^–^ + H_(aq)_^+^	(17)	79.87	14.00
Hydrogen peroxide acid constant:^7^	H_2_O_2(aq)_ ⇌ HO_2(aq)_^–^ + H_(aq)_^+^	(18)	66.70	11.69
Hydroperoxyl/superoxide radical acid constant:	HO_2(aq)_^•^ ⇌ O_2(aq)_^•–^ + H_(aq)_^+^	(19)	27	4.8
Hydroxyl radical acid constant:	HO_(aq)_^•^ ⇌ O_(aq)_^•–^ + H_(aq)_^+^	(20)	67	11.7

a We assume the standard state of all gaseous species is the solvated molecules in equilibrium with corresponding molecules in the gaseous state under standard conditions (298.15 K, 1 bar), X_(aq)_ ⇄ X_(g)_, and Δ_r_ *G* ^o^ = 0.00 kJ mol^–1^. Reaction free energies are for the reactions as written.

b Standard Free Energies of Formation used to calculate this table (and associated references) are provided in the Supporting Information (Table S4).

The electrochemical results are summarized in Figure 4, providing potentials at which peroxide, hydroperoxyl/superoxide, and hydroxyl radicals can be produced electrochemically from either O_2_ reduction (below H_2_O/O_2_ line) or H_2_O oxidation (above H_2_O/O_2_ line). Figure 4 is useful in understanding the domains under which hydrogen peroxide and different ROS can be electrochemically produced. In this summary, we define the standard state for oxygen as 1 bar oxygen gas, and the oxygen in solution is in equilibrium with that standard state oxygen (the alternative approach sometimes used assumes the standard state is 1 mol kg^–1^ dissolved oxygen).

**Figure 4:**
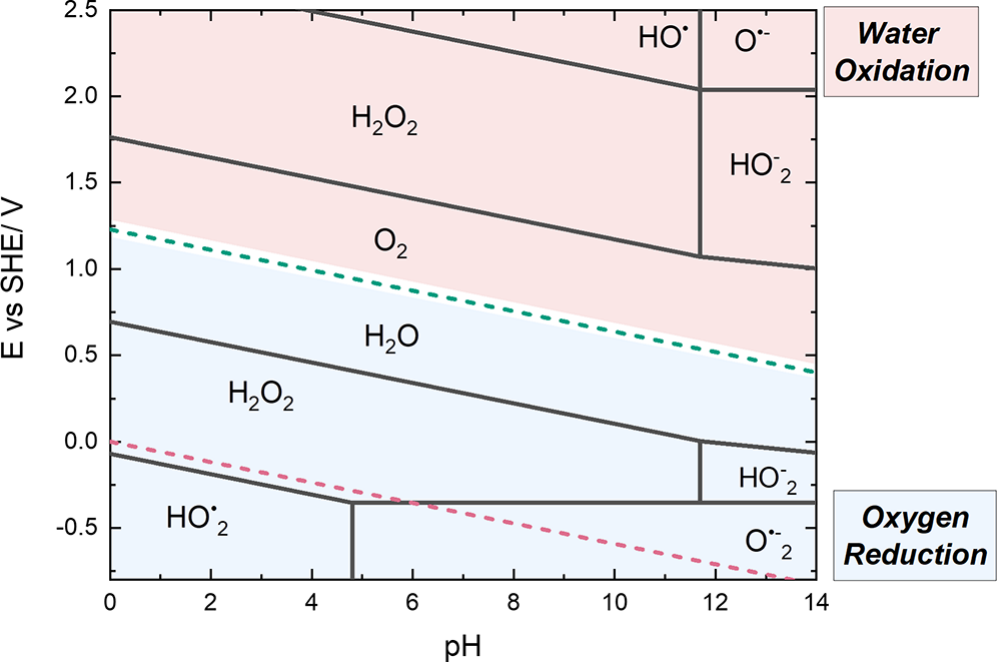
Diagram for the domains for oxygen reduction (blue region) or water oxidation (red region) to the respective species. Note that this is not a Pourbaix diagram, as the lines present the reactions involving either oxygen or water and not the adjacent phase. The green dotted line represents oxygen reduction to water (Table 2, eq 2); the red dotted line represents the hydrogen reaction (Table S1, eq 1). Calculated on the basis of all species in the standard state (activity of 1 mol kg^–1^ and aqueous solution is equilibrated at a pressure of 1 bar with gaseous species, 298 K).

### 2.2 Hydrogen Electrode

Under acidic conditions, the HOR/HER processes undergo the Volmer–Tafel and Volmer–Heyrovsky mechanisms,^26–28^ summarized in Table S1. The forward elementary reactions indicate the HER (Table S1, eq 1) for PEMWEs, while the backward reactions denote the HOR (Table S1, eq 2) for PEMFCs. In PEMWEs, the HER occurs at the cathode, where solvated protons diffuse from the bulk to the catalyst surface and then undergo electro-adsorption equilibrium to form adsorbed hydrogen atoms (Volmer step, Table S1, eq 5). These intermediates either proceed through the chemical Tafel step (Table S1, eq 3), evolving hydrogen on the catalyst surface, or undergo the electrochemical Heyrovsky step (Table S1, eq 4), in which the adsorbed hydrogen participates in a proton–electron concerted reaction via Eley–Rideal-type recombination.^29,30^ In PEMFCs, the HOR occurs at the anode, where hydrogen gas diffuses from the bulk and is adsorbed on the catalyst surface through either the Tafel or Heyrovsky step, followed by electro-oxidation via the Volmer step. The intermediate formation of adsorbed hydrogen atoms (H_ad_) appears as an important intermediate step in the chemical reduction of adventitious oxygen at the hydrogen electrode, as do appropriate adsorbed oxygen intermediates.

The hydrogen electrode would take part in formation of peroxide and hydroperoxyl species through reaction with adventitious oxygen which diffused across the membrane from the oxygen side. These species may be produced through either direct electrochemical reactions (Table 2, eqs 2 and 3) or indirect chemical reactions involving electrochemically produced H_ad_. These chemical reactions involve the adsorbed hydrogen described above and may directly produce a solvated hydroperoxyl radical and a free site (*).

21 $$H_{\mathrm{ad}}+O_{2\left(g\right)}\to HO_{2\left(\mathrm{aq}\right)}^{•}+*$$

The dissolved HO_2_^•^ may then undergo degradative reactions with the material of the electrolyzer/fuel cell or disproportionation to produce hydrogen peroxide (Table 2, eq 6), or it may adsorb to produce HO_2,ad_ and react with another adsorbed hydrogen atom to produce hydrogen peroxide:^8^

22 $$H_{\mathrm{ad}}+O_{2\left(g\right)}\to {\mathrm{HO}}_{2,\mathrm{ad}}$$

23 $$H_{\mathrm{ad}}+{\mathrm{HO}}_{2,\mathrm{ad}}\to H_{2}O_{2\left(\mathrm{aq}\right)}+*$$

The balance between these different processes is poorly understood but will depend on multiple factors such as the local oxygen concentration, the potential of the hydrogen electrode, etc.

### 2.3 Oxygen Electrode

#### 2.3.1 Oxygen Adsorption and Reduction Reaction in PEMFCs

Compared to the HER/HOR, the ORR and the OER are more complex and sluggish, making them the primary sources of inefficiency within fuel cells and electrolyzers. In microscopic descriptions of electrocatalysis systems, the electrode–electrolyte interface can be divided into three distinct regions: the electrolyte bulk, the diffusion layer, and the solvated electron-transfer layer. Before concerted electron–proton transfer occurs, oxygen is first adsorbed on the catalyst surface. Subsequent reactions and products are influenced by the type of adsorption. Three major types of oxygen adsorption have been considered: the Griffiths type, the Yeager type, and the Pauling type, as shown in Figure 5. The Pauling-type model is end-on adsorption, while the Griffiths and Yeager types represent side-on adsorption. Understanding oxygen adsorption is critical for comprehending peroxide formation at various catalytic sites on the electrode. The type of oxygen adsorption will depend on the nature of the catalyst involved in the reaction.

**Figure 5:**
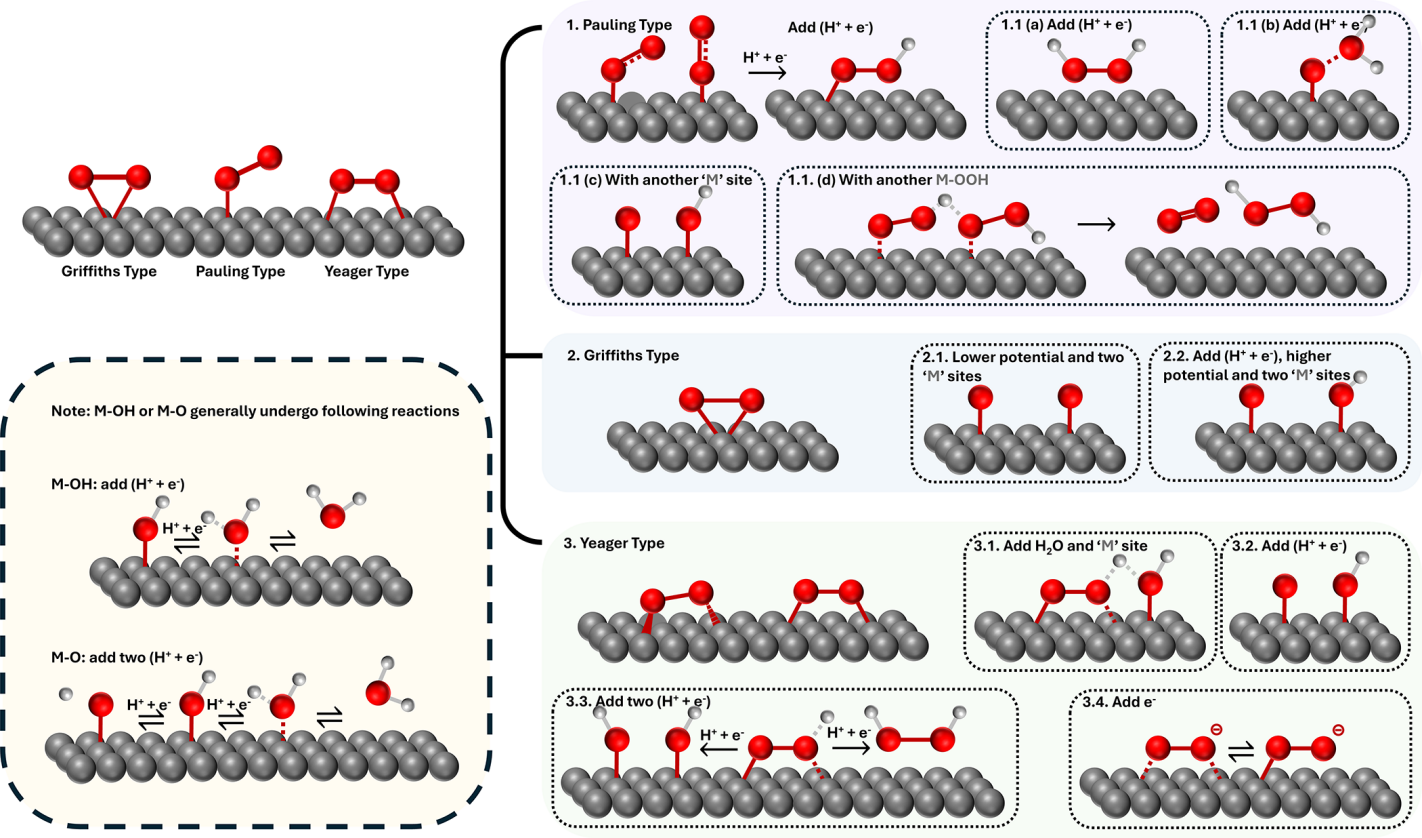
Three possible O_2_ adsorption models on the catalyst surface, where M represents an individual adsorption site. Individual colored panels show greater detail for the proton-coupled electron-transfer mechanism (yellow) and the (1) Pauling (purple), (2) Griffiths (blue), and (3) Yeager (green) sub-mechanisms.

In the PEMFCs, oxygen on the Pt/C surface is considered to adsorb in the Pauling (end-on) configuration on the metal site, as the side-on pathways are hindered.^31^ Because Pt provides a continuous surface with delocalized electronic states across its band structure, the formal oxidation state of the catalyst remains essentially unchanged during the ORR. Many of the steps involve a proton-coupled electron transfer (PCET)—that is, electron transfer coupled to the addition of a proton to the adsorbed species.^32^ In Pauling-type adsorption, the oxygen is adsorbed on the catalyst surface to form the M–OO species. Then, the superoxo species undergoes a concerted electron–proton transfer to form the M–OOH intermediate. Four possible reaction pathways for M–OOH species are summarized in Figure 5 (1.1­(a)−1.1­(d), shaded purple). This intermediate can either undergo an electrochemical concerted electron–proton transfer to selectively form undesired H_2_O_2_ (Figure 5, panel 1.1­(a)) or chemically react with neighboring M–OOH to produce H_2_O_2_ and O_2_ (Figure 5, panel 1.1­(d)),^33^ the adsorbed equivalent of the homogeneous disproportionation of solvated hydroperoxyl (Table 2, eq 16). Alternatively, the M–OOH can undergo a chemical step with the participation of an additional M site via dissociative adsorption to form M–O and M–OH (Figure 5, panel 1.1­(c)), eventually leading to H_2_O, through successive proton-coupled reduction of those species—shaded yellow in Figure 5. Otherwise, the concerted reaction occurs at the terminal oxygen atom to form water and M–O species through a PCET (Figure 5, panel 1.1­(b)),^34^ which then forms water through successive PCETs. DFT calculation^35^ suggested that the Pt itself is thermodynamically unfavored in producing peroxide, as the Pt–OOH bond is weaker than the Pt–O/Pt–OH bond, so once the Pt–OOH formed, it tends to quickly rearrange to the bridging chemisorbed state and favors O–O cleavage, proceeding via a 4-electron ORR route. Moreover, the as-produced peroxide can be electro-reduced rapidly on Pt or chemically disproportionated on Pt surfaces (Table 2, eq 13), both of which suppress the peroxide yield. However, the peroxide is still detectable on the Pt/C catalyst, and Figure 6 illustrates the sequence of reactions associated with the Pauling-type oxygen adsorption at the Pt active sites within the membrane. These lead to the release of H_2_O_2_, formation of ROS, and conversion of Pt catalysts into the soluble Pt^2+^ or Pt^4+^.^12^

**Figure 6:**
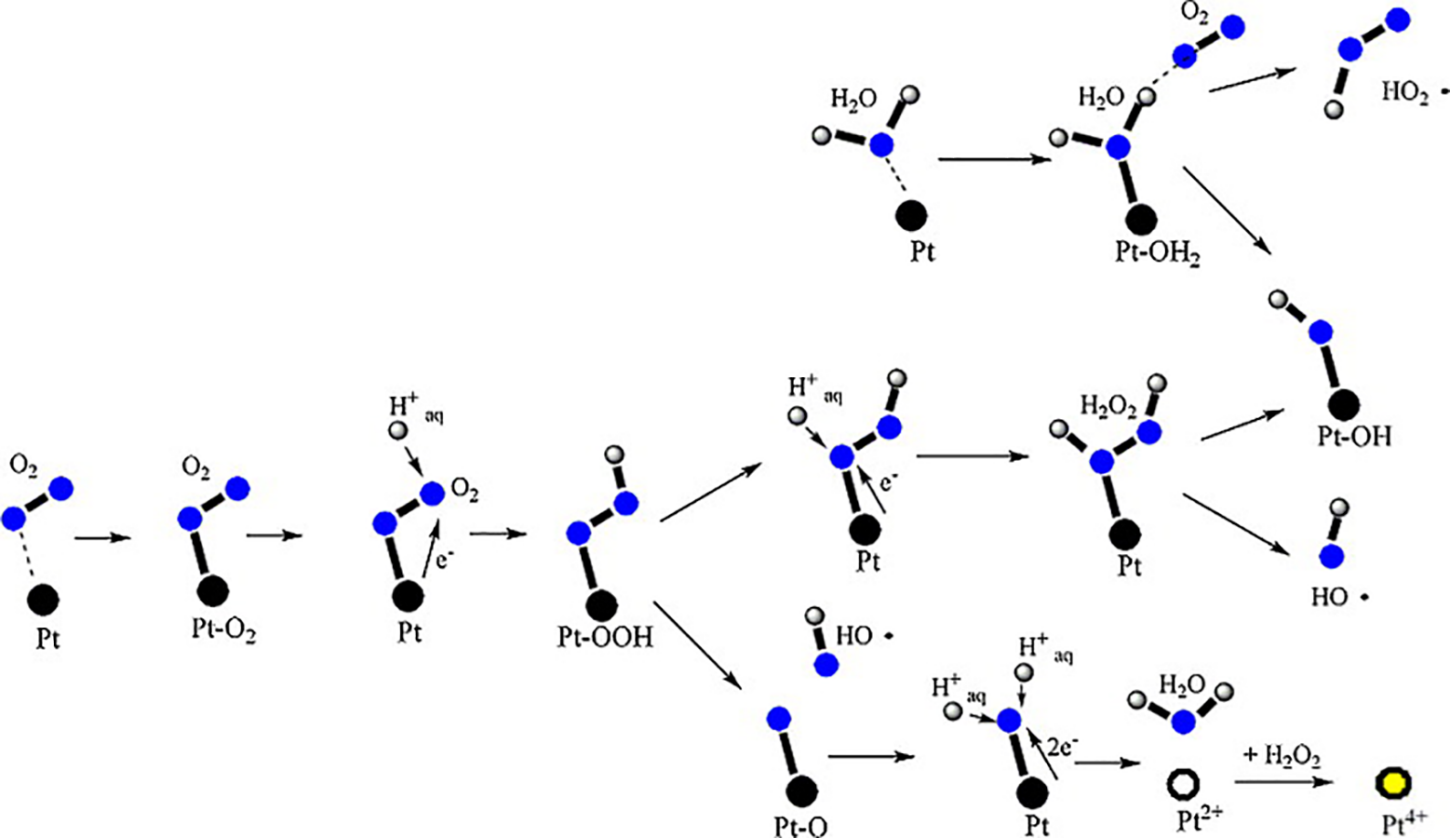
Radical formation and Pt dissolution in PEMFCs. Adapted with permission from ref 12. Copyright 2010 Elsevier.

Griffiths-type adsorption (Figure 5, panel 2), where oxygen is side-on adsorbed initially onto a single site, is not the dominant mode on Pt. The Griffiths model may be suitable for describing the single-atom catalyst active site (for instance, Fe-NC-type ORR catalysts) in which the catalytic site has a distinct oxidation state, where the atom is not embedded in a continuous electron cloud.^36,37^ The schematic reaction pathways and possible products of the Griffiths model are shown in Figure 5, panel 2, in the case where adsorption occurs on a continuous surface (i.e., not a single-atom catalyst). At a low enough potential (Figure 5, panel 2.1), the O–O bridge bond dissociates, and the oxygen atom forms the M–O adsorbate with adjacent catalyst sites, which is the rate-determining step. The potential dependence of this reaction is influenced by the layer close to the electrode surface or by the interaction between adsorbed anions and the adsorbed transition state complex.^20^ The formed M–O adsorbate then undergoes an electrochemical reaction to form water. At a more positive potential (Figure 5, panel 2.2), rather than first forming the M–O species, the direct proton–electron concerted reaction becomes the rate-determining step. Direct splitting via the Griffiths model is prone to the 4e^–^ pathway and forms water as the final product. This process skips the formation of the M–OOH adsorbate, thus avoiding the possibility of forming undesired H_2_O_2_.^36^

Wroblowa et al. provided a classical summary of the ORR pathway including the possibility for peroxide formation.^38^ The thermodynamic potential for the 4-electron ORR is 1.229 V, and the formation of HO_2,ad_ and desorption of OH_ad_ require an overpotential (η_theo_ ≈ 400 mV) to overcome the Gibbs free energy barrier before the reaction proceeds at useful rates, making the operational potential around 0.7 V.^31,39^ This means that the operational cathode potential is not so far from the potential at which hydrogen peroxide production is thermodynamically possible through the oxygen reduction process (Table 2, eq 2). For the cathode of fuel cells, common catalysts include Pt and its alloys, such as Pt_3_Co, Pt/Ni, and Pt_3_Zn.^40^ To optimize the catalytic specific surface area, the catalyst is usually dispersed on carbon, which has a large surface area. A step-by-step summary of ORR mechanisms is provided in Table S2.

In the PEMFC (and cathode of the PEMWE), the carbon support used for catalysts can interact with oxygen, especially supports with higher nitrogen contents and under alkaline conditions. On carbon materials, the formation pathways of adsorbed oxygen (O_ad_) and hydroxyl (OH_ad_) intermediates are less favorable (Figure 5, panel 3.2), and O–O bond scission is therefore disfavored. As a result, the 2-electron pathway becomes dominant,^20^ leading to the formation hydrogen peroxide and then of reactive radicals. Carbon then may simultaneously be attacked and degraded by those radicals. Several studies have shown that various carbon-based materials, such as carbon black, carbon nanotubes, and carbon felt,^41–43^ can serve as the potential source of H_2_O_2_ generation via the 2e^–^ ORR.

The mechanism of H_2_O_2_ generation on carbon was first proposed by Garten and Weiss and later reproduced by Yeager,^20^ Figure 5, panel 3. In this mechanism, oxygen first adsorbs side-on onto two different carbon sites, followed by the formation of O_2,ad_^–^ through the addition of 1 electron (Figure 5, panel 3.4). The proton source then rapidly protonates the O_2,ad_^–^, leading to the formation of HO_2,ad_, which subsequently undergoes a very fast disproportionation reaction to form oxygen and hydrogen peroxide.^44^ Additionally, O_2_ can also directly adsorb onto the carbon surface and then proceed through the following pathways: rapidly undergoing a PCET or interacting with the catalytic site and water (Figure 5, panel 3.1) to form HO_2,ad_, followed by a surface disproportionation reaction to produce H_2_O_2_. The adsorbed O_2_ can also undergo two PCET reactions to form hydrogen peroxide (Figure 5, panel 3.3, right).

#### 2.3.2 Water Oxidation Reactions in PEMWEs

The water oxidation reaction (WOR) can be categorized into three different pathways:^22,45–47^ 1e^–^ WOR, 2e^–^ WOR, and 4e^–^ WOR. The selectivity for the three different WOR pathways can be systematically categorized from the thermodynamics viewpoint via the standard potential of the associated products. The first step of all three WORs involves the formation of OH_ad_ by a PCET (Table S3, eq 18). With Δ_f_ *G*_OH_ad__ ≳ 2.38 eV, the OH_ad_ radical desorbs from the catalyst surface and the 1e^–^ WOR is favored (Table S3, eq 19). For Δ_f_ *G*_OH_ad__ ≲ 2.38 eV, the 2e^–^ and 4e^–^ WOR paths are favored. The competition between these two oxidations depends on the Δ_f_ *G*_O_ad__ (Table S3, eq 23). With Δ_f_ *G*_O_ad__ ≳ 3.53 eV, the 2e^–^ WOR is preferred.^25^ A summary of the individual steps for the WOR is provided in Table S3.

In acidic media, OER at an iridium-based PEMWE anode exhibits an onset overpotential of 250–300 mV.^48^ The mechanistic steps for the OER are the same as for the ORR in principle, running the reverse of that process. Although the exact mechanism is not fully understood, two generally accepted mechanisms are the adsorbate evolution mechanism (AEM) and the lattice oxygen participation mechanism (LOM), shown in Figure 7a,b. These two mechanisms are similar, with the mechanistic difference between them being whether the active site is a metal atom or an oxygen vacancy in the lattice. In the AEM, the OER is related to the adsorption energy between the intermediates and the adsorption site. In the first elementary reaction, the hydroxyl group (−OH) is adsorbed on the catalyst surface, forming a Cat–OH bond. In the second step, the Cat–OH is deprotonated. By addition of another water, Cat–OOH is formed (step 3), and at the final step (step 4), oxygen gas is formed from the Cat–OOH. The LOM mechanism is less reliant on the adsorption energy. The metal oxide is involved in the reaction, where lattice oxygen is part of the cycle via O–O coupling. Oxygen gas is then released, leaving a lattice oxygen vacancy that can be refilled by water.

**Figure 7:**
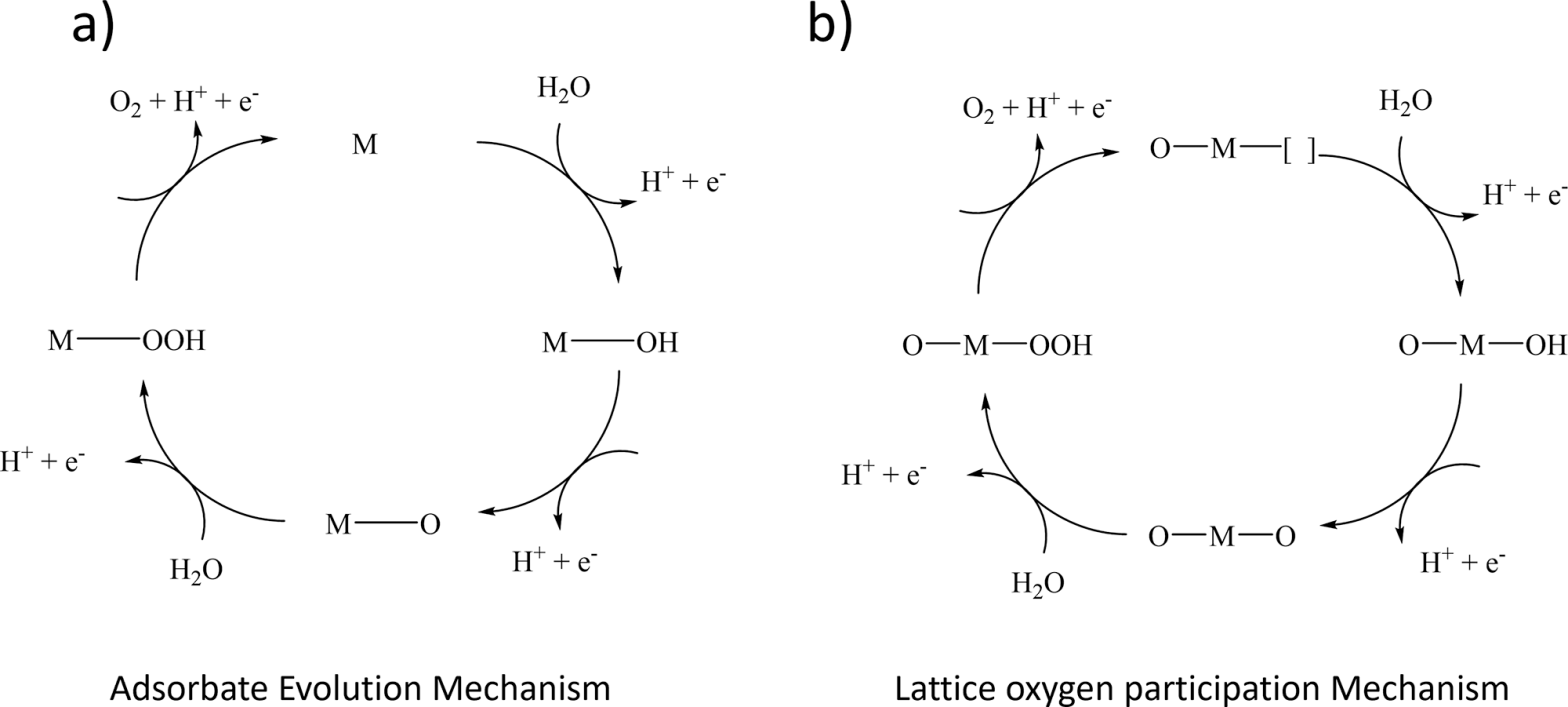
(a) AEM in an acidic environment. (b) LOM in an acidic environment. M represents the metal active site, and the square brackets indicate the lattice oxygen vacancy.

For PEMWEs, while RuO_2_ is the most active catalyst for the OER, it is less stable than IrO_2_ for the OER in an acidic environment. Direct water oxidation to produce peroxide in the PEMWE is thermodynamically unfavorable at the anode side due to the high potential of that reaction (Table 2, eq 4); however, there are some suggestions that hydrogen peroxide may be detected during water oxidation on iridium oxide.^22^

#### 2.3.3 Combining Oxygen Reduction and Evolution with the Hydrogen Reaction in Chemical and Electrochemical Processes

In this review, compared to the classical Wroblowa ORR and AEM/LOM OER mechanisms, we updated the reaction pathways to explicitly include both chemical and electrochemical peroxide formation as well as the production of superoxide/hydroperoxyl as an intermediate, Figure 8. We also include the hydrogen reaction. In PEMFCs, oxygen typically undergoes a direct 4e^–^ ORR to form H_2_O via either an associative or a dissociative mechanism at the cathode.^14,31,38^ We want to highlight how these reactions, which typically occur on different electrodes in a PEMFC/PEMWE, can also occur on the same catalyst particle when that catalyst particle is in a mixed gas (H_2_/O_2_) environment. The precise adsorbed species and mechanisms vary with different catalysts and reaction environment, as described above. In Figure 8, we represent the balance of the reactions between oxygen evolution (left-hand side) and reduction (right-hand side) and between peroxide formation (top) and decomposition (bottom). We show both chemical and electrochemical pathways, including the processes associated with mass transport of species away from the catalytic surface. An important intermediate is adsorbed hydrogen, which is involved with several chemical reactions. The direction of the reactions is biased to the left or right depending on whether the operation is electrolytic (electrolyzer, left) or galvanic (fuel cell, right).

**Figure 8:**
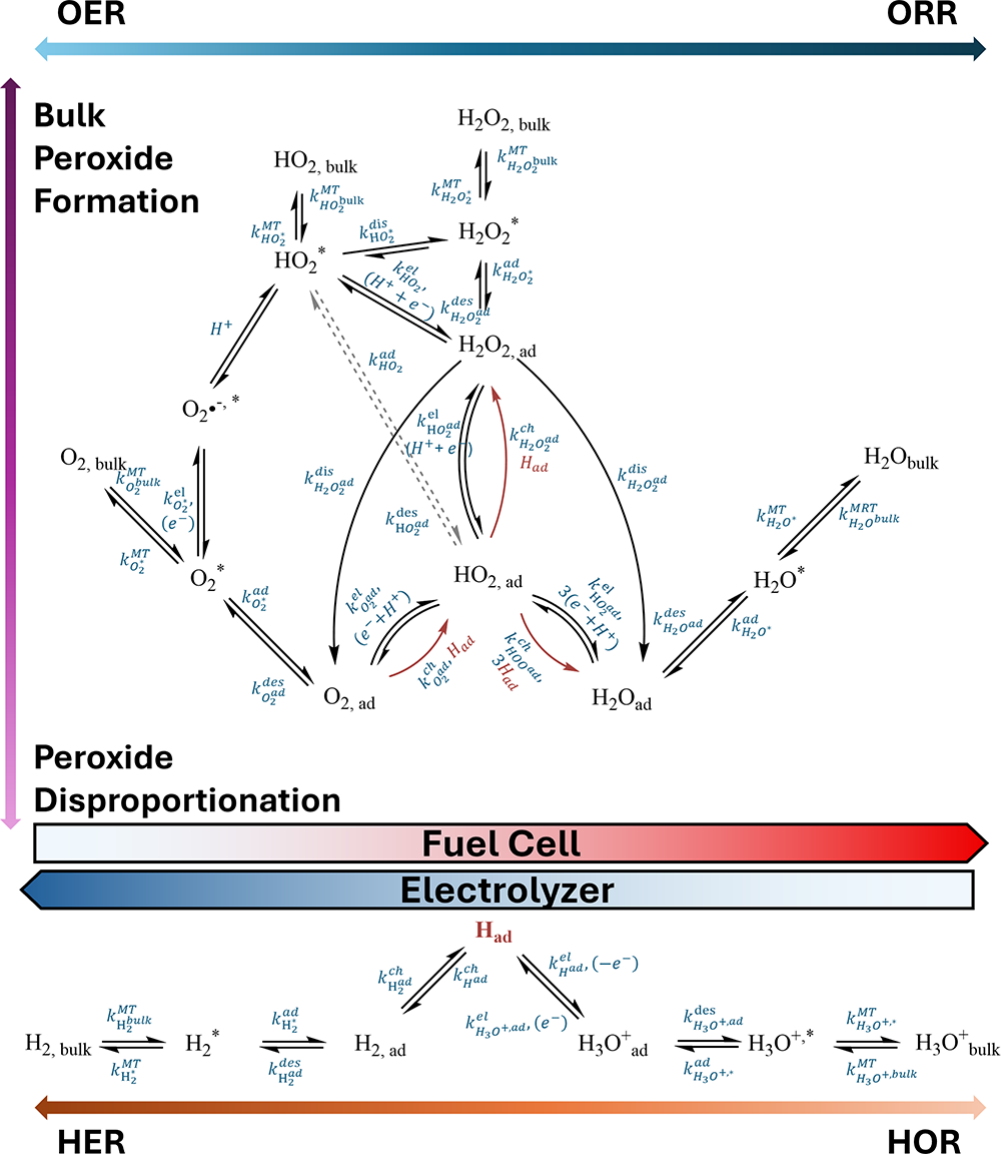
Oxygen evolution/reduction reaction pathways: k^el^ – the electrochemical pathway, k^ch^ – the electrochemical pathway, k^MT^ – mass transport kinetics, k^ad^ and k^des^ – adsorption and desorption kinetics. * represents more than one water molecule distance from the surface, and subscript “ad” represents the adsorbed species. For the sake of clarity, a number of intermediates (O_ad_, OH_ad_) are not shown. All non-adsorbed species are in hydrated form (subscript “(aq)”).

## 3 Conditions for Gas Crossover and H_2_O_2_ Generation in a MEA

Gas crossover is primarily influenced by the properties of the membrane and the operational conditions. A commonly used electrolyte material in both PEMFCs and PEMWEs is a PFSA polymer, for example, Nafion, Aquivion, or 3M polymer. A sulfonated tetrafluoro­ethylene-based fluoropolymer copolymer functions as a proton conductor and stabilizing agent. It comprises a rigid perfluorinated (polytetrafluoro­ethylene, PTFE) backbone, an intermediate flexible fluorinated ether side chain, and a hydrophilic sulfonated acid group, which is referred to as the hydrated ionic cluster region.^49^ Its fully fluorinated structure resists oxidative attack, while the sulfonated acid groups facilitate proton exchange through percolation networks and rapid water transport via an interconnected cluster–network model.^50^

In a single cell, gas movement is driven by the need to equalize the concentrations (partial pressure) on both sides of the membrane. The rate of this movement is dependent on the solubility of the gas in the membrane material and the rate at which it can diffuse or permeate through the membrane. The mathematical relationship describing the gas permeability rate (*N*_*i*_) of the species *i* through the perfluoro­sulfonic acid membrane, such as Nafion, is represented below, where *H*_*i*_ is the solubility coefficient, *p*_*i*_ is the gas pressure, *l* is the membrane thickness, and *D*_*i*_ is the effective diffusion coefficient, with subscript “h” representing high and subscript “l” representing low concentrations.^51–53^

24 $$N_{i}=D_{i}\frac{H_{i}^{h}p_{i}^{h}-H_{i}^{l}p_{i}^{l}}{l}$$

In porous media, the effective diffusion coefficient (*D*_*i*_) is often approximated by using porosity–tortuosity relationships. However, this framework is not generally appropriate for PFSA membranes, which are phase-separated ionomers rather than conventional porous solids. In PFSA membranes, dissolved gases can permeate through both the solid polymer phase and the water-rich domains, and both the hydration state and the morphology of the polymer contribute to *D*_*i*_.^54^ Experimental results using Nafion indicate that thicker membranes reduce gas crossover,^55^ as expected from eq 21. However, a thicker membrane increases the ion transport distance, raising ohmic resistance. Hence, there exists a trade-off between performance and gas crossover. Thinner membranes exhibit lower specific resistance (lower internal resistance), require less material, and thus reduce production costs. Moreover, they improve the water back-transport and distribution. However, thinner membranes result in higher gas crossovers (which result in lower efficiencies and make safety aspects more critical) and reduced mechanical stability. The study by Schalenbach et al. showed that, as the membrane thickness decreases, the efficiency loss due to hydrogen crossover becomes more significant than the gains achieved from reduced resistance.^56^

Further studies^58–60^ suggest that gas flux through Nafion membranes is not entirely dependent on membrane thickness, with concentration gradients due to supersaturation being a major driving force of gas transport. Peripheral components and configurations, such as the gas diffusion layer (GDL), flow field plate, and cell compression rate, can influence the gas crossover. For instance, a previous study indicates that compressing the GDL slightly impacts gas crossover.^61^ The cartoon on the left-hand side of Figure 9 shows the hydrogen profile under two conditions: the first in which the hydrogen is supplied in the cathode compartment and removed in the anode compartment but no electrochemical reaction occurs (static case, solid line); the second in which electrochemical production of hydrogen leads to supersaturation in the cathode catalyst layer but the pressure of hydrogen is kept under the same conditions within the cathode compartment as the static one (dynamic case, dotted). The gas concentration differs between the layers due to phase solubility differences and changes in the partition coefficients. Under operating conditions (dashed line), where gas evolution occurs in the PEMWE, the local gas concentration in the catalyst layer can exceed the solubility limit, resulting in gas supersaturation. Schalenbach et al. demonstrated that, in addition to gas transport limitations, such supersaturation can increase the interfacial gas partial pressure and raise the dissolved gas concentration via Henry’s law, thereby enhancing the driving force for diffusion-driven crossover across the membrane,^56^ as shown by the dotted line in Figure 9.

**Figure 9:**
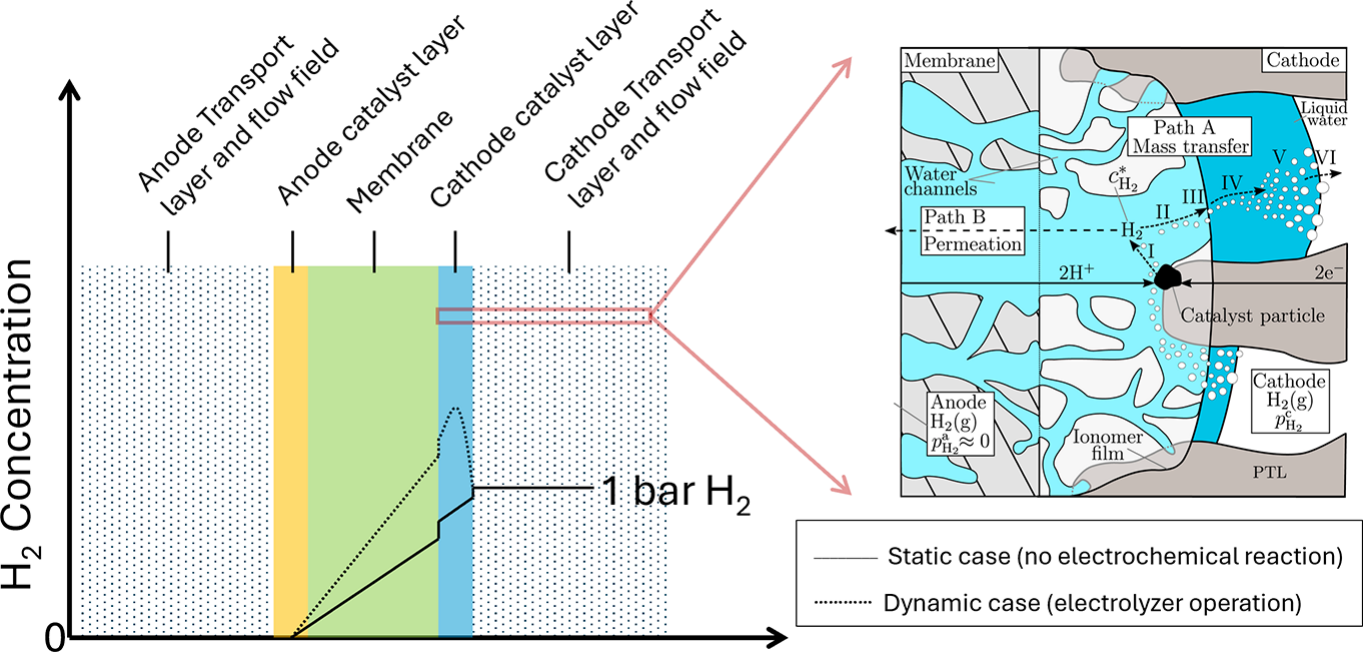
Comparison of hydrogen concentration profiles of PEMWEs across the membrane under diffusion-limited electrolyzer operation conditions (dotted line) and where there is no electrochemical reaction (solid line) in which the hydrogen perssure on the cathode side is fixed at 1 bar. Inset: Schematic illustration of H_2_ supersaturation within the ionomer film on the cathode side (not to scale). Mass transfer (Path A) is divided into several stages: I, molecular H_2_ desorbed from the catalyst surface; II, movement through the ionomer; III, transfer across the interface into liquid water; IV, passage through the water; V, bubble formation and growth at the nucleation site; and VI, transfer across the interface into the gas phase. The catalyst is shaded in black, and the porous transport layer (PTL) is shaded in dark gray. Reproduced with permission from ref 57. Copyright 2017 Elsevier.

Increasing the water content in the membrane at constant temperature raises the permeability coefficient (*k*_*i*_) for both H_2_ and O_2_.^52,55,62^ This increase is primarily attributed to a rise in the *D*_*i*_, while the *H*_*i*_ remains relatively unchanged, and rearranging eq 24 provides^52^

25 $$k_{i}=D_{i}H_{i}$$

Sakai et al.^63^ explained this phenomenon using a cluster-network model. In the membrane, water acts as a plasticizer. Under low relative humidity (RH), Nafion contains only small amounts of water, which is absorbed within the isolated clusters. Because these clusters are not interconnected, the limited water present has a negligible effect on gas diffusion through the membrane. Of course, for electrolyzers, the presence of liquid water adjacent to the membrane provides high water content in the membrane. In contrast, fuel cells are strongly affected by the water content (RH%) in the adjacent gas phases. With high RH, narrow channels between the clusters become water-filled, creating a continuous network. With increased water uptake, the distance between the polymer chains expands, reducing the van der Waals forces between them. This change in the structural dimensions can enhance gas transportation via diffusion-driven gas crossover by providing more pathways through the water-filled channels.^64^

Temperature affects the diffusion coefficient, as diffusion is an activated process. At higher operating temperatures, molecular movement accelerates, leading to faster diffusion and a higher permeability coefficient, which ultimately results in greater gas crossover, as illustrated in Figure 10 for Nafion.

**Figure 10:**
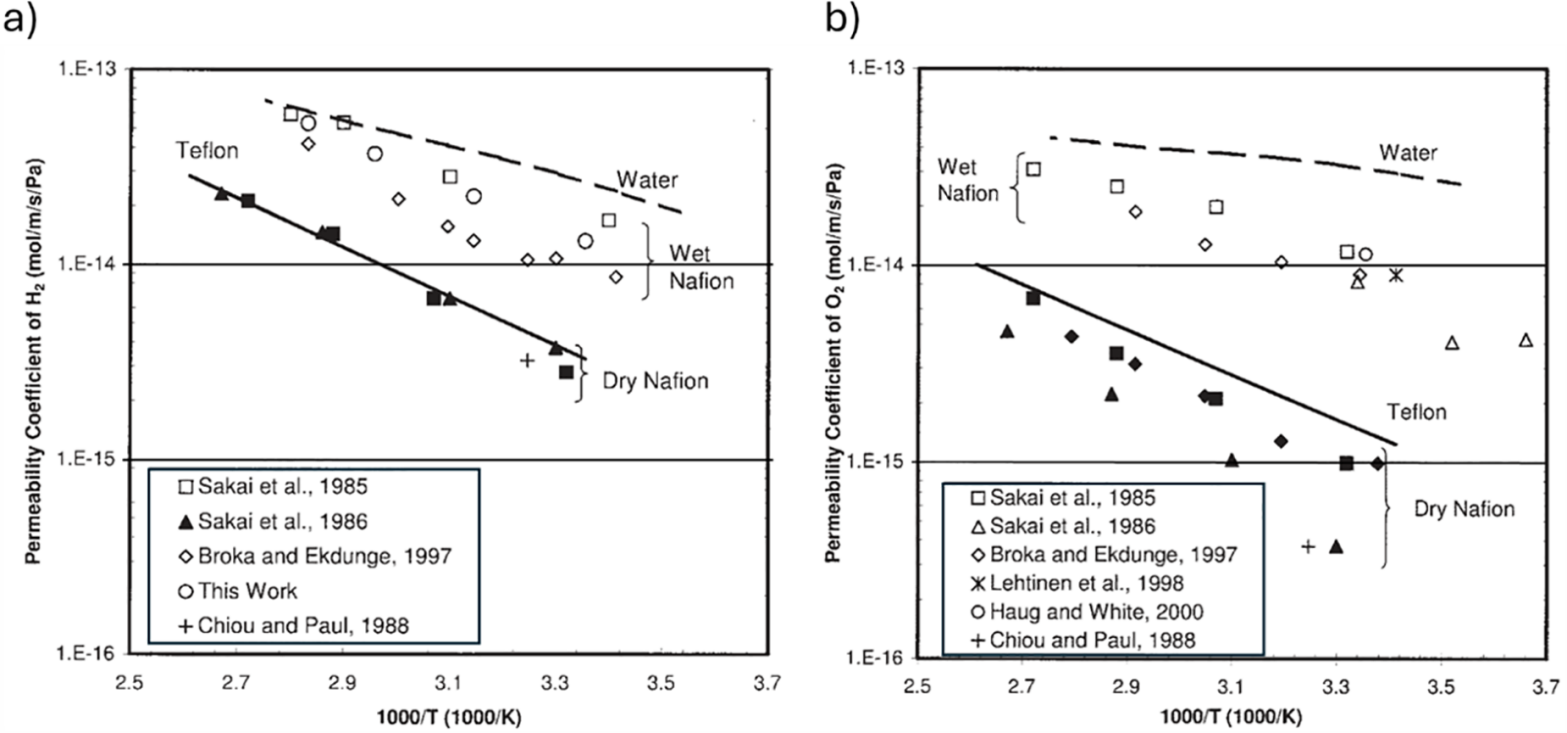
Hydrogen (left) and oxygen (right) permeability coefficients of Nafion 117; for comparison, ▲ and △ represent the values for Nafion 125, solid lines show the reported permeability coefficients for Teflon, and dashed lines show the reported permeability coefficients for water. Reproduced with permission from ref 52. Copyright 2006 Wiley.

While elevated pressure can improve fuel cell performance, it also increases gas crossover. For the intact membrane, the crossover is generally diffusion-dominated. The contribution of differential pressure-driven (Darcy-type) transportation is insignificant in the pressure ranges that are relevant to PEM devices when using perfluoro­polymer-type materials.^54^ By Henry’s law, the concentration of a dissolved species is directly related to its partial pressure in the gas phase, and the partial pressure of the gas at a specific electrode equals the total pressure minus the water vapor pressure and any diluent.^53,65^ Consequently, a larger partial pressure gradient across the membrane produces a larger concentration gradient and, by Fickian diffusion, drives greater diffusive crossover flux from the high to the low partial pressure side, eq 24.^52,55,66–68^

Fuel cells typically employ markedly thinner membranes, whereas electrolyzers are operated at substantially higher pressures with thicker membranes. Hence, the resultant diffusion-driven crossover fluxes need not differ greatly. In a PEMWE during operation, molecular hydrogen initially evolved and desorbed from Pt/C catalyst sites subsequently dissolves in water, covering the ionomer.

These dissolved hydrogen molecules then encounter irregularities on the electrode surface and begin to nucleate heterogeneously—a conceptual illustration of such interfacial phenomena is shown in the inset of Figure 9.^57^ However, before nucleation occurs, hydrogen concentration in water can become supersaturated, as suggested by Vogt et al.^69^ This occurs because the critical heterogeneous supersaturation limit is lower than the theoretical homogeneous supersaturation limit predicted by Henry’s law.^67^ The dissolved hydrogen can take either path A shown in in the insert of Figure 9, being released to the porous transport layer, or alternatively path B, entering the membrane and contributing to the gas crossover. Hydrogen supersaturation is more pronounced at higher current densities.^57,70^ A similar process can occur at the anode of the PEMWE, increasing the oxygen flux across the membrane.

Electro-osmotic drag affects the water distribution in the membrane and can indirectly influence the gas crossover by advective transport of the gas within the transported water. As illustrated in Figure 1, protons moving from the anode to the cathode drag water molecules along with them, also transporting dissolved gases across the membrane. This effect is particularly pronounced in humidified systems or those with liquid water. In PEMFCs, dragging water from the hydrogen side to the oxygen side can lead to issues such as cathode flooding, which may impede gas diffusion and reduce cell efficiency. In PEMWEs, electro-osmotic drag increases oxygen crossover from the oxygen electrode to the hydrogen electrode, causing recombination on the cathode (and peroxide generation) and oxygen to mix with product hydrogen. The oxygen drag flux density (Φ_O_2__^drag^) equation^56^ provides an estimate of the increased crossover of oxygen due to this mechanism:

26 $$\Phi_{O_{2}}^{\mathrm{drag}}=\frac{j}{F}\xi \frac{p_{O_{2}}^{\mathrm{an}}S_{O_{2}}}{c\left(H_{2}O\right)},,\xi =0.0134\frac{1}{K}\times T+0.03$$

where *j* is the current density, *F* is the Faraday constant, ξ is the experimentally determined temperature-dependent dimensionless drag coefficient, *p*_O_2__^an^ is the oxygen partial pressure, *S*_O_2__ is the oxygen solubility, and *c*(H_2_O) is the amount of water molecules at the anode. In a PEMWE, electro-osmotic drag can also mitigate hydrogen crossover to the anode to some extent by potentially transporting some of the dissolved hydrogen back to the hydrogen electrode.^53,71–73^

In summary, the crossover in PEMFCs and PEMWEs is primarily governed by diffusion and electro-osmotic effects. During operation, the selection of RH, pressure, operating temperature, membrane thickness, and transport layer compression all contribute to the gas crossover, and in the case of PEMWEs, supersaturation can enhance these gas crossover phenomena. Gas crossover results in the aggravation of membrane degradation and cell performance loss. Details of the influence of gases crossover on H_2_O_2_ formation in MEAs are discussed in the next section.

## 4 Influence of Gas Crossover on H_2_O_2_ Production in MEAs

### 4.1 Influence of Gas Crossover

#### 4.1.1 Oxygen Crossover to the Hydrogen Electrode

Due to the membrane’s permeability, oxygen diffuses from the oxygen electrode to the hydrogen electrode. The reaction steps leading to hydrogen peroxide formation due to oxygen crossover to the hydrogen electrode are embodied in all the reactions shown in Figure 8, as both the hydrogen and oxygen reactions are occurring on one catalyst particle (rather than being separated on different electrodes). Direct electrochemical reduction of oxygen to hydrogen peroxide (Table 2, eq 2, and Figure 2a) is possible on the platinum hydrogen catalyst particles present in the hydrogen electrode, and this is typically considered the most common mechanism. However, because of the high coverage of H_ad_ on the catalyst surface (disfavoring oxygen adsorption) and the low potential of the electrode, other pathways also become possible.

Possible processes leading to hydrogen peroxide generation are illustrated in the cartoon in Figure 11a, leading to the incomplete electrochemical reduction of oxygen (Table 2, eq 2) or the direct chemical combination reaction between H_2_ and O_2_, which results in peroxide generation (Table 2, eq 10). The presence of impurities such as adsorbed chloride enhances peroxide release by facilitating O–O bond cleavage.^74^ As shown in in Figure 11a, oxygen reacts with chemisorbed H_ad_ to produce HO_2,ad_ (eq 22, Figure 2 b) and then hydrogen peroxide (eq 23).^8^

**Figure 11:**
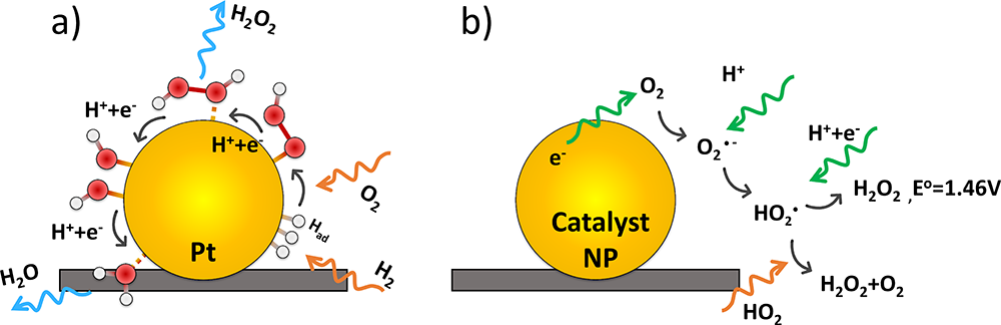
(a) Molecular-level H_2_O_2_ formation mechanism: oxygen crossover to the hydrogen-covered platinum electrode. (b) Outer-sphere peroxide formation mechanism: reduction of oxygen to superoxide radical species followed by disproportionation.

An alternate electrochemical pathway for peroxide formation, depicted in Figure 11b, involves an outer-sphere electron-transfer reaction, first undergoing an outer-sphere 1-electron ORR to form a superoxide radical (O_2__(aq)_^•–^). Under acidic conditions, this is followed by fast proton transfer from the surrounding solution to produce its protonated form, the hydroperoxyl radical (HO_2(aq)_^•^, p*K*_a_ = 4.8). Under acidic conditions, this reaction will occur at a lower potential than the direct formation of the hydroperoxyl radical, HO_2(aq)_^•^ (Table 2, eq 3). The hydroperoxyl radical is highly reactive and may degrade the local ionomer and catalyst support (see below). It may undergo a fast disproportionation reaction with another HO_2(aq)_^•^ to produce hydrogen peroxide and oxygen (Table 2, eq 16).^20,75^ Alternatively, it may undergo a second outer-sphere electron transfer to produce the hydrogen peroxide anion (HO_2_^–^, Table 2, eq 6), followed by subsequent protonation to form hydrogen peroxide.

For electrolyzers, oxygen crossover to the hydrogen electrode is generally considered less detrimental to safe operation, as the stoichiometric amount of oxygen migrating to the hydrogen side is only half that of the reverse direction and PEMWEs tend to operate with large pressure differentials from hydrogen to oxygen sides. In PEMWEs, the operating pressure at the oxygen electrode is close to atmospheric, reducing the comparative flux of oxygen permeating through the membrane.^76^ Nevertheless, studies have shown that, in the presence of Pt at the hydrogen electrode, peroxide formation can occur.^74^

#### 4.1.2 Hydrogen Crossover to the Oxygen Electrode

Hydrogen crossover to the oxygen electrode catalyst, which under operating conditions will be covered with oxygen, would result in peroxide formation chemically, as shown in Figure 2d, with mechanisms discussed in Section 2.3.1. Alternatively, permeated hydrogen may react electrochemically with oxygen to produce peroxide, as illustrated in Figure 2e. The distinction between these electrochemical and chemical pathways remains poorly understood and should be investigated in the future.

In electrolyzers, hydrogen crossover to the oxygen electrode raises safety concerns, as it can approach the lower flammability limit (LFL), particularly under high-pressure and low-current-density operating conditions. This issue has received increasing research attention in recent years.^53,57,77^ Most studies have focused on reducing the hydrogen concentration in oxygen and improving Faradaic efficiency, while membrane degradation near the oxygen electrode has been largely overlooked. Theoretical production of hydrogen peroxide at the oxygen electrode of an electrolyzer via eq 4 shown in Table 2 has been recently reported^10^ when the oxygen electrode is operating under high operating potentials (peaking at 1.8 V), as shown in Figure 2 f).

#### 4.1.3 Oxygen Crossover and Hydrogen Crossover within the Membrane

Addition of hydrogen/oxygen recombination catalysts (typically Pt/C) to the membrane either as a band or homogeneously deposited in the membrane is used as a method to reduce hydrogen crossover in PEMWEs by allowing the hydrogen present in the membrane to recombine with oxygen, generating water, heat, and possibly peroxide, Figure 2c.

Although such an approach is not typically used within fuel cells, bands of platinum catalyst can form spontaneously. Dissolution of platinum predominantly at the oxygen electrode in PEMFCs, as illustrated in Figure 12, can lead to the formation of platinum deposits within the membrane. As previously mentioned in Figure 6, one of the Pt-related degradation mechanisms involves the diffusion of Pt ions into the ionomer phase due to the concentration gradient and eventually into the membrane. When Pt ions encounter hydrogen (resulting from gas crossover), they may be reduced back to metallic Pt, forming a band of metallic Pt in the membrane near the oxygen electrode of the fuel cell, as shown in Figure 2. The band location within the membrane is influenced by the partial pressure of gases.^79^ The mathematical representation of the band location^80^ under diffusion-limited conditions is provided below:

27 $$\delta_{b}=\frac{2c_{O_{2}}^{0}D_{O_{2}}}{c_{H_{2}}^{0}D_{H_{2}}+2c_{O_{2}}^{0}D_{O_{2}}}\delta_{m}=\frac{2D_{O_{2}}}{D_{H_{2}}\left(\frac{p_{H_{2}}^{0}H_{O_{2}}}{p_{O_{2}}^{0}H_{H_{2}}}\right)+2D_{O_{2}}}\delta_{m}$$

where δ_b_ and δ_m_ are the distance between the Pt band and the hydrogen electrode and the membrane thickness, respectively, and *c*_O_2__^0^ and *c*_H_2__^0^ are oxygen/hydrogen concentrations at the boundary between the membrane and the cathode/anode, respectively. *D*_O_2__ and *D*_H_2__ are diffusion coefficients of oxygen and hydrogen in the membrane. *p*_H_2__^0^ and *p*_O_2__^0^ are the oxygen/hydrogen partial pressure at the boundary between the membrane and the cathode/anode, respectively. For PEMWEs, migration of both Pt and Ir takes place within the membrane. The Pt band was discovered in the membrane around the hydrogen electrode similar to but not as significant as in PEMFCs, whereas the Ir band was found also near the hydrogen electrode due to Ir dissolution and redeposition.^81^

**Figure 12:**
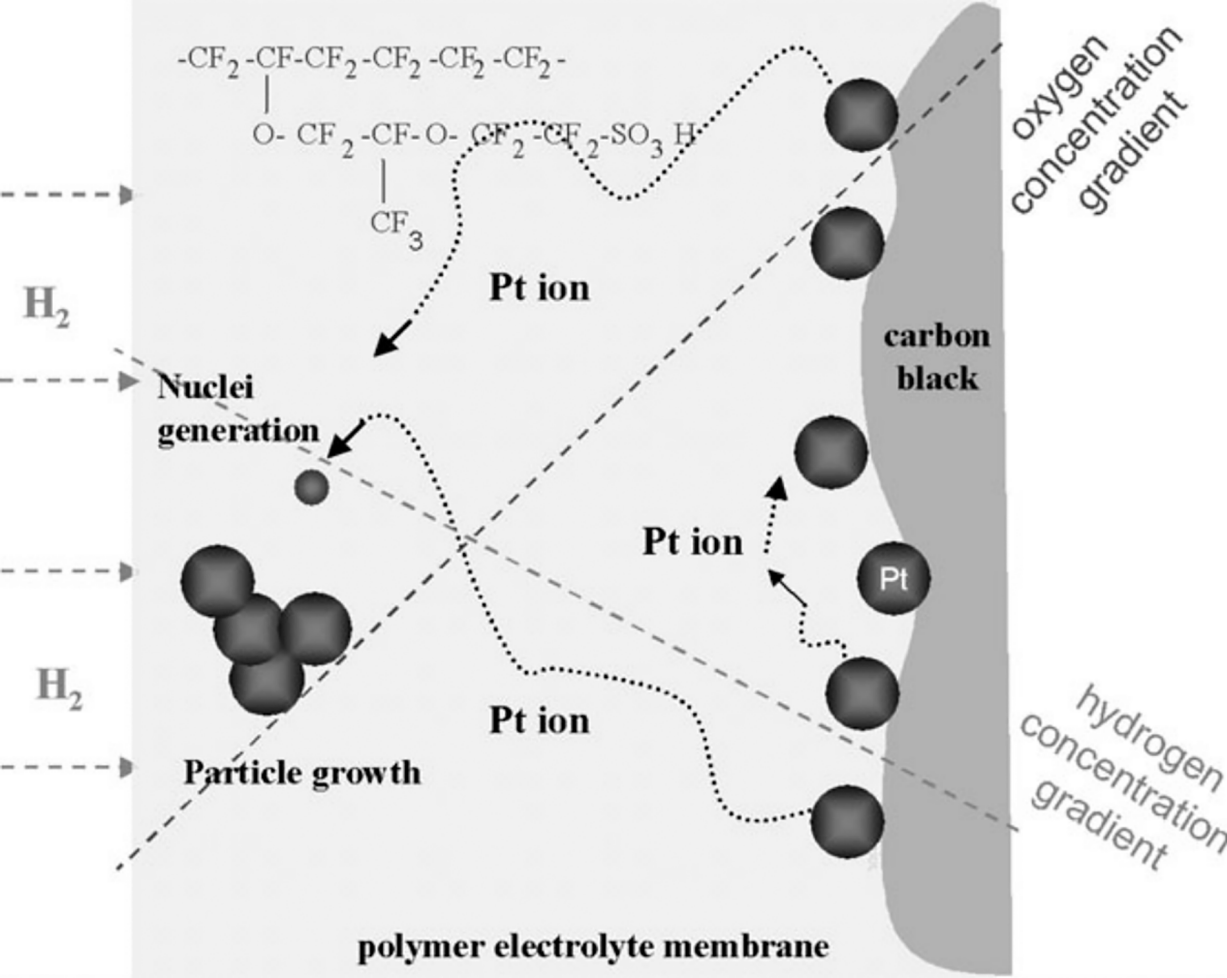
Model of Pt dissolution and deposition in the membrane. Reproduced with permission from ref 78. Copyright 2006 Royal Society of Chemistry.

The rate of the Pt band formation (via reaction with H_2_) in the membrane is about 1/1000th of the H_2_ crossover rate. This is because the reaction between Pt^2+^ and H_2_ is less competitive than the reaction between H_2_ and O_2_ on the Pt band, as the Pt band predominantly serves as a catalyst.^79^ Further research has shown that, for higher Pt ion concentrations in the MEA, despite variations in hydrogen crossover or diffusion distance, the Pt ion flux and its deposition in the membrane remain largely unchanged.^82^ Band formation signifies a loss of catalyst and electrochemical surface area at the electrode, resulting in reduced durability and membrane degradation. Additionally, the Pt band obstructs proton transport channels, decreasing the membrane conductivity.

However, the impact of the Pt band formation remains a topic of debate. Metallic Pt particles act as sinks by effectively quenching radicals, and H_2_O_2_ can undergo autocatalytic decomposition in the presence of Pt to form water and oxygen. The dispersed catalyst sites in the membrane are also capable of forming H_2_O_2_ (shown in Figure 2c) and catalyzing direct radical formation, leading to oxygen reduction. The model developed by Gummalla et al.^83^ suggested that the net membrane degradation rate is governed by the balance between radical generation and radical quenching, which depends strongly on Pt particle size and spacing (Pt “band” morphology), as well as on local H_2_/O_2_ crossover and membrane hydration conditions. The advantages and disadvantages of Pt band formation have been thoroughly evaluated by Ren et al.^80^ In addition to peroxide formation, gas crossover and recombination can lead to the direct chemical combination of H_2_ and O_2_, producing heat and water without generating electricity. This process results in significant hot spot formation and localized fuel starvation.^52,80,84^

### 4.2 Effect of H_2_O_2_ and Reactive Oxygen Species (ROS)

#### 4.2.1 Membrane

From a thermodynamic viewpoint and in order of driving force, ROS may be produced chemically through Fenton-type reactions (considered the major pathway), electrochemically, or through direct chemical processes involving hydrogen peroxide or hydrogen and oxygen.

Homolysis of hydrogen peroxide into two neutral radical species (Table 2, eqs 14 and 15) is a thermodynamically unfavorable processes. Homolysis of hydrogen peroxide to two hydroxyl radicals (Table 2, eq 14) is known to occur photolytically or radiolytically, and in those cases, the energy required to break the peroxide bond is provided by a high-energy photon. Thermally driven homolysis may occur at high temperatures (several hundred degrees Celsius) but is not likely to be appreciable under electrolyzer/fuel cell operating conditions. The same product radicals (to hydroxyl, or to hydroperoxyl and hydrogen radicals) can also, in principle, be produced through combination of hydrogen and oxygen gases (Table 2, eqs 11 and 12), and although both of the reactions are 134 kJ mol^–1^ more favorable than producing these radicals from hydrogen peroxide (i.e., the formation energy of hydrogen peroxide, Table 2, eq 10), they still remain thermodynamically unfavorable processes. Direct combination of the gases to two hydroxyl radicals (Table 2, eq 11) requires only 53 kJ mol^–1^ free energy but would be a kinetically difficult reaction to achieve as it involves simultaneously breaking of both the oxygen and hydrogen bonds. In contrast, direct combination of hydrogen and oxygen to hydroperoxyl and hydrogen radicals (Table 2, eq 12) does not involve breaking the oxygen bond (just the hydrogen bond), but in free solution the free energy is quite unfavorable due to the high free energy of formation of the hydrogen radical. More likely is the involvement of a surface-adsorbed hydrogen atom (e.g., on the surface of the platinum catalyst) reacting with an oxygen atom in solution (eq 21).

Direct electrochemical production of hydroxyl and hydroperoxyl radicals via water oxidation (Table 2, eqs 5 and 8) is too energetically unfavorable to occur in a water electrolyzer or fuel cell under usual operating conditions (*E* ^o^ = 2.73 and 1.66 V, respectively). In contrast, reduction of oxygen to the hydroperoxyl radical (Table 2, eq 3, *E* ^o^ = −0.07 V) is possible, in principle, as especially the cathode in an electrolyzer would be polarized within this potential region during normal operation. The oxygen source would be associated with crossover. Thus, the possibility of direct formation of hydroperoxyl radicals must be considered as a distinct possibility.

The as-generated H_2_O_2_ may undergo the Fenton reaction to form ROS, which is considered the major pathway for the production of these species. This process is catalyzed by a trace amount of transition metal impurities M^*n*+^ originating from cell or stack corrosion. These impurities, including but not limited to Fe^2+^, Cu^2+^, and Ti^3+^, are likely to remain trapped in the membrane.^16,80^ A requirement for the Fenton catalysts is that they have relatively low-lying redox potentials (*E*_M^*n*^/M^*n*+1^_^o^ = 0.771, 0.159, and 0.1 V for Fe^2+^, Cu^+^, and Ti^3+^, respectively). Cation impurities can form cation–water clusters, which affect the membrane’s water uptake ability and, thus, conductivity.^85,86^ More seriously, cation impurities can facilitate the decomposition of H_2_O_2_, producing hydrogen radicals (H_(aq)_^•^), hydroxyl radicals (HO_(aq)_^•^), and hydroperoxyl radicals (HO_2(aq)_^•^), as shown in Table 3.^80,87–89^

Under neutral pH, Cu^2+^ exhibits high catalytic activity for the Fenton reaction, while Fe^2+^ has the highest ROS production rate.^90^ Moreover, research conducted by Danilczuk et al.^91^ demonstrated that, in PEMFCs, oxygen crossover to the anode or hydrogen crossover to the cathode can also lead to the direct production of HO^•^ radicals on the catalyst surface, as shown in Table 3, eq 33. Subsequent studies using the fluorescence probe indicated that the concentration of HO^•^ radicals at the hydrogen electrode was higher than at the oxygen electrode.^8,92^

The radicals generated are potent oxidizing agents that can attack the PSFA membrane, resulting in the production of F^–^ and low-molecular-weight PFSA species. The half-life of HO_2,(aq)_^•^ is about 1.5 μs,^19^ so due to its short half-life, the ROS would only react with nearby components. Four primary membrane attack mechanisms are summarized by Zaton et al.^16^ and illustrated in Figure 13 and Table 4. Such decomposition reactions are liable to be faster in membrane materials composed of non-perfluorinated components.

**Table 3:** Summary of Reactive Oxygen Species (ROS) Generation Reactions with Rate Constants and Standard Free Energy of Reaction (Δ_r_ *G*°)^19^

**Reaction**		**Rate constant (M^–1^ s^–1^)**	**Δ_r_ *G* (kJ mol^–1^)**
M_(aq)_^*n*+^ + H_2_O_2(aq)_ + H_(aq)_^+^ → M_(aq)_^(*n*+1)+^ + HO_(aq)_^•^ + H_2_O_(l)_	(28)	63^a^	–2^a^ ^,^ ^b^
M_(aq)_^(*n*+1)+^ + H_2_O_2(aq)_ → M_(aq)_^*n*+^ + HO_2(aq)_^•^ + H_(aq)_^+^	(29)	4 × 10^–5^ ^a^	67^a^ ^,^ ^b^
HO_(aq)_^•^ + H_2_O_2(aq)_ → HO_2(aq)_^•^ + H_2_O_(l)_	(30)	2.7 × 10^7^	–122^a^ ^,^ ^b^
HO_(aq)_^•^ + H_2__(g)_ → H_(aq)_^•^ + H_2_O_(l)_	(31)	4.3 × 10^7^	–40^b^
H_(aq)_^•^ + O_2__(g)_ → HO_2(aq)_^•^	(32)	1.2 × 10^10^	–216^b^
H_2__(g)_ + O_2__(g)_ → 2HO_(aq)_^•^ (at the catalyst surface)	(33)	—	53^b^

a For *M*_(aq)_^*n*+^ = Fe^2+^.

b These values have been calculated using the standard free energy of formation of the respective species provided in the Supporting Information (Table S4).

**Figure 13:**
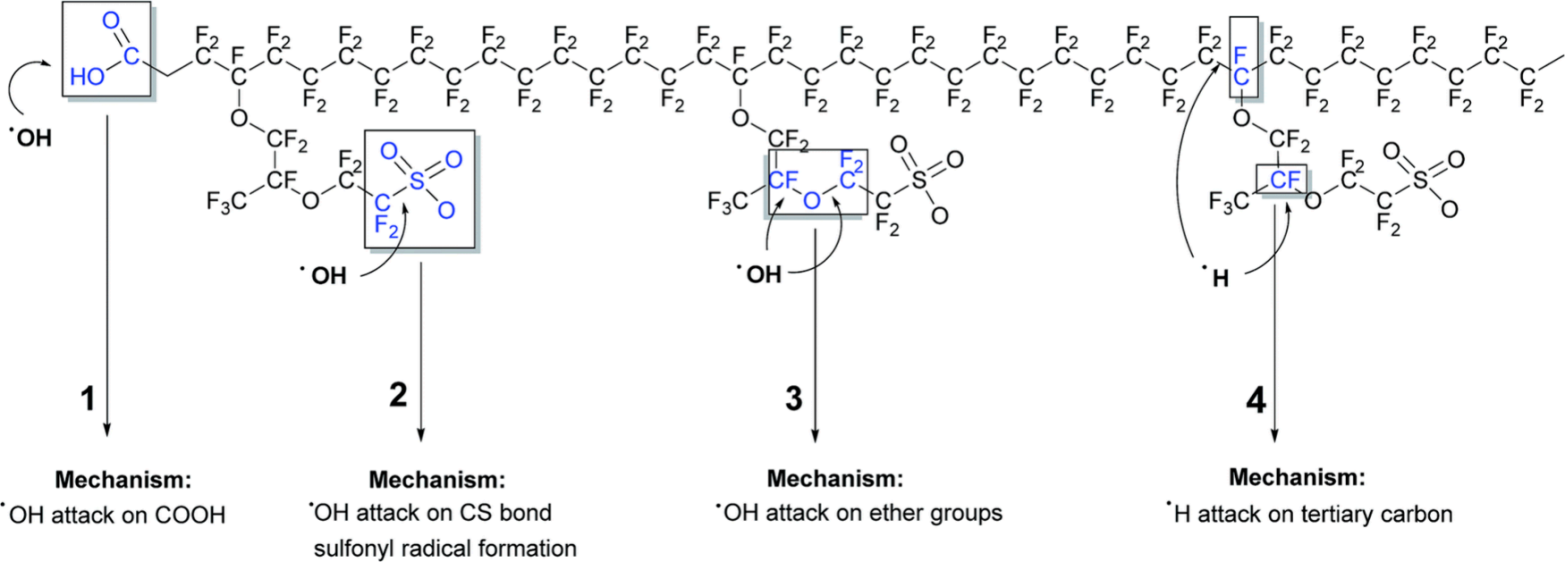
Schematic illustration of four radical attack mechanisms on the Nafion polymer structure. Reproduced with permission from ref 16. Copyright 2017 Royal Society of Chemistry.

**Table 4:** Reaction Rates for Different Radical Attack Mechanisms, with Activation Energy (Δ*E*_a_) Calculated Using DFT and Corrected for Solvation^93–97^

**Reaction**	**Rate constant (M^–1^ s^–1^)**	**Activation energy Δ*E*_a_(kJ mol^–1^)**
HO^•^ attack terminal COOH	10^6^	93.6
HO^•^ attack on C–S bond	—	92.6
HO^•^ attack on ether group	3.7 × 10^6^	135.1
H^•^ attack on tertiary carbon	—	96.5

The first mechanism is usually referred to as the unzipping mechanism, where the HO_(aq)_^•^ radical attacks the terminal carboxylic acid groups (−OOH) and produces CO_2_ and HF. In the second mechanism, the HO_(aq)_^•^ radical attacks the C–S bond of the sulfonic acid moiety (−SO_3_H), resulting in SO_3_ and HF release. The third mechanism is the HO_(aq)_^•^ radical attacking both sides of the ether group, referred as the ether cleavage mechanism; the mechanism and products were proposed by Ghassemzadeh et al.^98^ The fourth mechanism involves the H_(aq)_^•^ radical attacking the tertiary carbon, resulting in chain scission or side chain decomposition. Among these four mechanisms, the end-group unzipping mechanism is regarded as the predominant degradation pathway. Nevertheless, as with any covalent bond, the C–S linkage can undergo cleavage under sufficiently aggressive conditions; however, under typical fuel-cell or electrolysis operating regimes, such cleavage is not generally favored.^99,100^ The radical attack on the membrane results in worsened conductivity as well as fluoride ion emission and membrane thinning.

#### 4.2.2 Carbon-Based Materials

Carbon-based materials are frequently used in fuel cells and electrolyzers, serving as catalyst supports, microporous layers (MPLs), and GDLs. Conductive carbon materials such as carbon black are commonly employed as catalyst supports and in MPLs in PEMFCs and as cathodes in PEMWEs. As described in Section 2.3.1, oxygen reduction on carbon leads predominantly to hydrogen peroxide. Carbon black, which is amorphous and composed of microcrystalline primary particles, contains surface defects. The unsaturated atoms with mixed valence at the edges of carbon planes make these regions particularly reactive and prone to corrosion.^101^ Carbon degrades irreversibly under operational conditions, forming CO_2_ as shown in eq 34.^101,102^

34 $$C+2H_{2}O_{\left(l\right)}\to {\mathrm{CO}}_{2\left(g\right)}+4H_{\left(\mathrm{aq}\right)}^{+}+4e^{-},,E^{{}^{\circ}}=0.207\;V$$

Operating conditions such as pressure and temperature can be varied to evaluate the gas crossover. By connecting the cell to a gas chromatograph, the crossover mechanism can be characterized in detail.^53^ This method is particularly effective for evaluating the performance of the recombination catalysts.

Electrochemical carbon corrosion occurs at the fuel cell cathode, leading to a loss of the active surface area and electrical conductivity. An electron spin resonance (ESR) experiment by Endoh et al.^103^ indicated that, apart from oxidation-induced corrosion, carbon is also vulnerable to radical attack, with a probable mechanism illustrated in Figure 14. Furthermore, during startup and shutdown, oxygen crossover from the anode to the cathode in PEMFCs exacerbates damage via the “reverse current” mechanism.^104^

**Figure 14:**
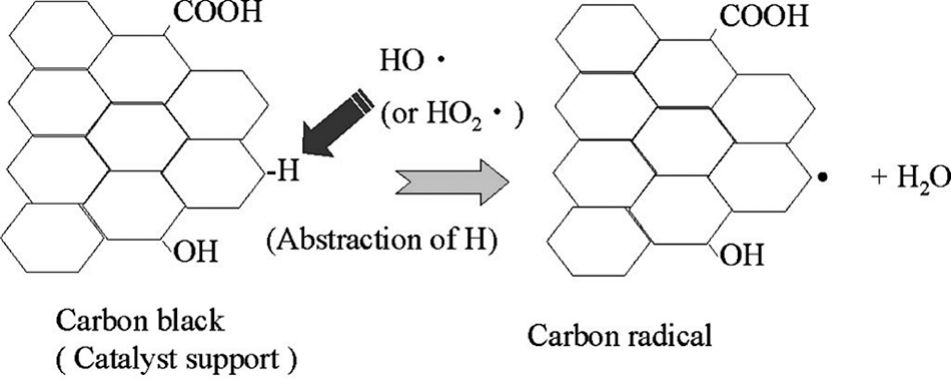
Carbon radical formation mechanism. Adapted with permission from ref 103. Copyright 2004 The Electrochemical Society.

## 5 H_2_O_2_ Detection Protocols

The extent of H_2_O_2_ production in PEMFCs or PEMWEs can be detected by using chemical or electrochemical methods.

### 5.1 Chemical Detection

Chemical methods primarily depend on specific reactions initiated by peroxide or ROS to generate measurable signals. While these methods are straightforward and easy to implement, they may lack real-time monitoring capabilities compared with electrochemical techniques.

#### 5.1.1 Direct Detection of H_2_O_2_

Volumetric titration involves using a titrant to determine the unknown concentration of H_2_O_2_,^45,105^ where common titrants include KMnO_4_ and Ce­(SO_4_)_2_—for example, 2Ce_(aq)_^4+^ + H_2_O_2(aq)_ → 2Ce_(aq)_^3+^ + 2H_(aq)_^+^ + O_2(g)_, where the solution changes color from yellow to colorless at the end point. This method offers a rapid means of quantifying peroxide formation, requiring neither calibration nor specialized equipment. The detection range is 30–300 mM H_2_O_2_. However, in PEMFCs and PEMWEs, the peroxide present at the gas outlet is often highly diluted, and accuracy can be compromised due to ambiguity in end point determination.

Colorimetric quantification via spectrophotometric measurement (UV–vis) offers much higher sensitivity toward peroxide detection, giving a lower minimum detection limit^45^ (0.01–0.12 mM H_2_O_2_). Color change is caused by oxidizing or reducing a transition metal by H_2_O_2_. The peroxide concentration is linearly related to the absorbance at a specific wavelength, so that the peroxide concentration can be determined quantitatively. An example of colorimetric quantification uses 0.1 M TiOSO_4_, where TiOSO_4(aq)_ + H_2_O_2(aq)_ → Ti­(O_2_)­SO_4(aq)_ + H_2_O_(l)_, λ_max_ = 407 nm.^106^ For H_2_O_2_ at the micromolar level, Amplex Red is commonly used in neutral or slightly acidic conditions in the presence of horseradish peroxidase (HRP). The Amplex Red molecule is oxidized by peroxide to the highly fluorescent product resorufin.^107^ The accuracy of these methods is intrinsically reliant on the precision of the established H_2_O_2_ calibration concentration, and color instability of the oxidized species can result in inaccuracies.

#### 5.1.2 Detection of Reactive Oxygen Species (ROS)

The 2,2′-azino-bis­(3-ethylbenzothiazoline-6-sulfonate) (ABTS)–peroxidase system, measured via UV–vis^108,109^ spectroscopy, is commonly used to evaluate the concentration of ROS. In this method, ABTS reacts with ROS to produce the ABTS^•+^ radical, with a characteristic spectrum shift from λ_max_ = 340 nm (ABTS) to λ_max_ = 414 nm (ABTS^•+^). This quantitative approach is rapid, simple, and highly sensitive, with a detection limit of 0.06–0.25 μM. However, the ABTS^•+^ radical decays over time, requiring measurements to be performed with a specific time window to avoid inaccuracies. In addition, due to the nonspecific nature of the reaction, this method cannot distinguish the precise source of ROS.

#### 5.1.3 Indirect Detection of Degradation Products

The above methods are ex situ. In situ monitoring allows continuous observation, whereas ex situ analysis requires periodic extraction of samples for analysis using specialized equipment. Fluorine emission rate (FER) analysis^110^ is a sensitive and accurate semicontinuous method for assessing the chemical durability of membranes by detecting F^–^ ions that originate from the degradation of fluorinated polymer membranes via HF dissociation in the water flowing from the cell. This approach enables quantitative analysis of fluorine-containing ions without dismantling a single cell, allowing real-time monitoring and the simultaneous detection of other degradation byproducts. FER measurement also serves as an indirect indicator of gas crossover, particularly when crossover occurs without significant membrane degradation, although its applicability is limited to fluorinated membranes. Recently, Marocco et al.^111^ developed an automated online FER measurement coupled with ion chromatography, enabling semicontinuous monitoring of fluoride ion release. They further established a correlation between F^–^ concentration and water conductivity, providing a more feasible route for real-time monitoring; however, if the catalyst exhibits instability, the conductivity-based reading may be misinterpreted. Operating conditions such as pressure and temperature can be varied to evaluate gas crossover.

### 5.2 Electrochemical Detection

#### 5.2.1 Half-Cell Setup

Electrochemical methods offer the opportunity to monitor peroxide levels in the electrochemical system both in situ and ex situ.

The rotating ring-disk electrode (RRDE) technique, as shown in Figure 15a, employs a working electrode consisting of a central disk electrode surrounded by a concentric ring electrode and is commonly used as an in situ half-cell method for peroxide detection during electrocatalyst development.^14,115,116^ At the disk electrode, H_2_O and H_2_O_2_ are generated electrochemically, while at the ring electrode, H_2_O_2_ is consumed electrochemically. The average number of electrons (*n*) and peroxide yield (*Y*_peroxide_) are calculated from the ring (*I*_R_) and the disk (*I*_D_) with collection efficiency (ε) using the relations shown below:

35 $$n=\frac{4I_{D}}{I_{D}+\frac{I_{R}}{\varepsilon }}$$

36 $$Y_{\mathrm{peroxide}}=\frac{\frac{2I_{R}}{\varepsilon }}{I_{D}+\frac{I_{R}}{\varepsilon }}\times 100\%$$

**Figure 15:**
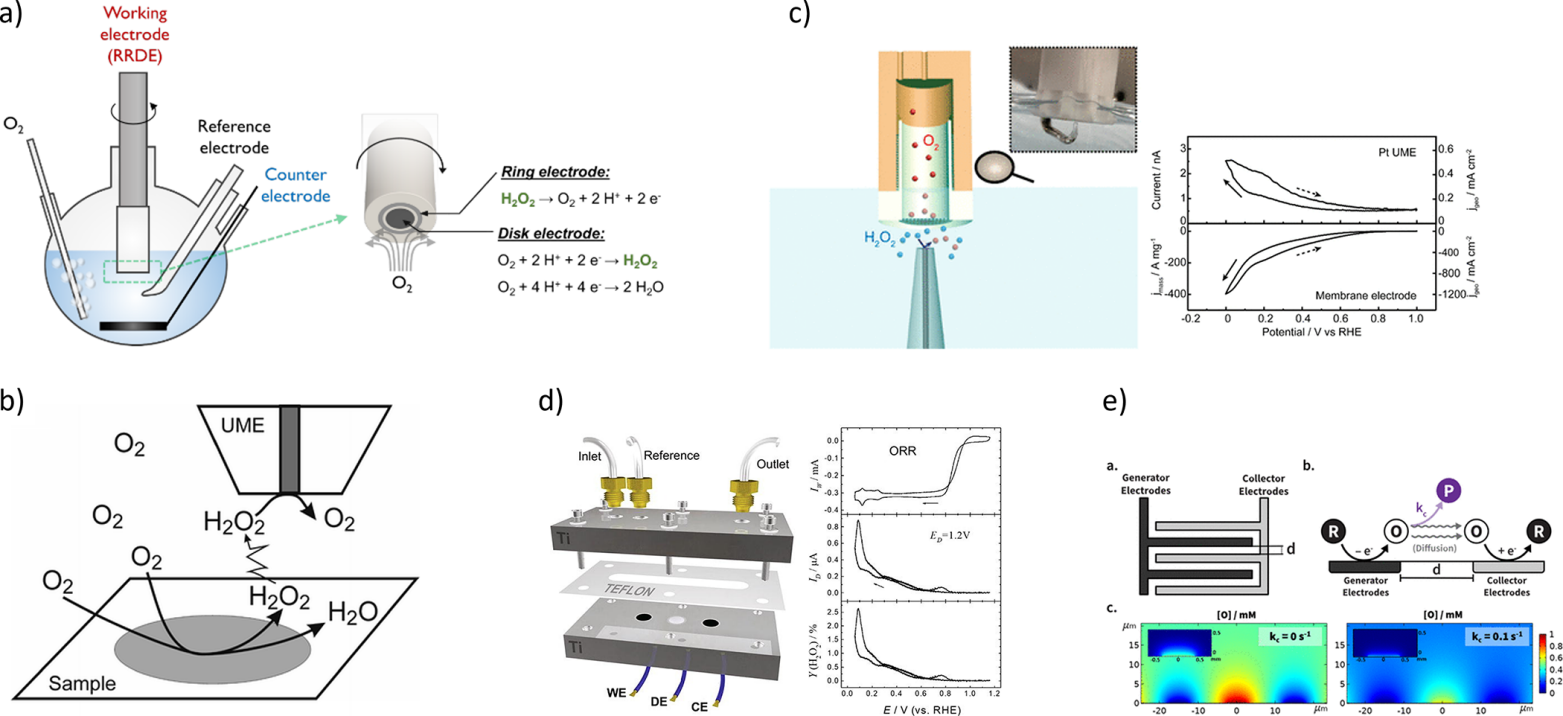
(a) Schematic setup of RRDE for peroxide detection. Reprinted with permission from ref 14. Copyright 2018 American Chemical Society. (b) Schematic illustration of the SECM operation in the pulsed substrate generation/tip collection (SG/TC) mode. Reproduced with permission from ref 112. Copyright 2008 American Chemical Society. (c) (Left) Gas-accessible membrane electrode (GAME) coupled with an ultramicroelectrode (UME), set up for peroxide detection, with the GAME producing peroxide and the Pt UME placed close to the GAME (separation ca. 100 μm) to oxidize the as-produced peroxide. (Right) Pt/C-deposited Au/PCTE recorded at 10 mV s^–1^ in 1 M HClO_4_ (bottom) and the corresponding UME response (top), held at 1.0 V. Scan directions are indicated by the arrows: forward, solid; backward, dashed. Reprinted with permission from ref 113. Copyright 2020 American Chemical Society. (d) (Left) Schematic diagram of the DDECFC construction. (Right) Cyclic voltammograms of the working electrode (Pt/Vulcan), the corresponding current at the detector electrode, and the yield of H_2_O_2_ at O_2_-saturated 0.5 M H_2_SO_4_, with an electrolyte volumetric flow rate of 20 μL/s and scan rate of 5 mV/s. Reproduced with permission from ref 114. Copyright 2021 Elsevier. (e) a. IDA geometry, b. work flow of generation/collection mode of IDA, and c. concentration profiles of oxidized species O in the absence and presence of a chemical reaction with a constant gap width (*d*) at the electrode surface. Reproduced from ref 115. Copyright 2023 Pence et al., available under a CC-BY-NC-ND 4.0 license.

This in situ monitoring method enables simultaneous detection and quantification of H_2_O_2_ generation as well as the ability to probe reaction pathways and catalyst efficiency, providing valuable insights into electrochemical reaction mechanisms. However, this technique is limited by low mass transport and current density, typically 2–3 orders of magnitude lower than those of MEAs. Due to the differences in geometry from fuel cells, the oxygen diffuses through the electrolyte rather than arriving in gaseous form from the opposite side. Furthermore, this method is unable to operate efficiently at elevated temperatures and pressures, and thus it cannot accurately represent real-world conditions. The OER operates under very harsh conditions, where high potential (up to 1.9 V) can oxidize and etch electrode materials, causing electrode degradation.

Similar to RRDE, scanning electrochemical microscopy (SECM),^112,117–119^ as shown in Figure 15b, employs a substrate electrode designed to generate water and peroxide, while a tip electrode, typically a Pt ultramicroelectrode (UME), is used to detect the resulting peroxide signal through oxidation of peroxide to water. The effective detection region is located around the UME, making this technique particularly suitable for probing specific areas. As SECM is very sensitive to separation between the tip and the substrate, such a system is particularly powerful for mapping local peroxide formation with the tip.^120^ However, SECM instrumentation is costly, and precise control of the electrode–electrode distance is challenging. Moreover, the geometry of the UME, including the tip diameter and effective probe dimensions, can markedly influence the measured data. As UMEs are commonly fabricated in-house, reproducibility between electrodes is often limited, posing challenges for obtaining consistent experimental results. Therefore, computational simulations are commonly employed to minimize the discrepancies arising from geometric effects. Operation at elevated temperatures and pressures is not possible with current systems.

Kucernak et al.^113^ developed a gas-accessible membrane electrode (GAME) for an in situ half-cell method offering 2–3 orders of magnitude higher mass transport than conventional RRDE setups, as shown in Figure 15c. In this configuration, various catalysts can be deposited onto a gold-coated microporous polycarbonate film of defined dimensions with gas flowing through the porous, thin-film electrode (12 μm thick electrode structures). The GAME is positioned above a UME, enabling the formation of a three-phase interface. By immersion of the UME in the electrolyte in a face-up “submarine” orientation, peroxide can be detected across a wide potential range. This technique is functionally analogous to SECM but requires significantly less expensive instrumentation. Furthermore, it enables the examination of electrocatalytic processes without the mass transport limitations commonly encountered in other techniques (RRDE, SECM, etc.). However, similar to SECM, as the fabrication of UMEs varies, the analytical model is required to accurately quantify the peroxide formation.

In addition to the above methods, novel microfluidic half-cell setups, such as the double-disk-electrode channel flow cell (DDECFC) and the interdigitated array electrode (IDA), are being unitized to investigate peroxide formation. The DDECFC,^114,121^ as shown in Figure 15d, consists of an electrolyte stream flowing over an upstream working electrode and a downstream detector electrode, with diffusion occurring between the adjacent electrode strips. In this configuration, oxygen reduction currents are measured at the upstream working electrode, while hydrogen peroxide oxidation currents are detected at the downstream electrode. As an improved version of the conventional channel flow cell, the DDECFC enables H_2_O_2_ detection under elevated temperature (up to 110 °C) and pressure (up to 2.5 bar) and allows precise control of H_2_ or O_2_ concentrations in the electrolyte. However, wall and edge effects can lead to a reduced current density along the electrode edges (*z*-coordinate) and intensified current density in the flow direction (*x*-coordinate).

The IDA,^122–124^ as shown in Figure 15e, is also commonly implemented in microfluidic (μ-Fl) systems, similar to DDECFC but with generator and collector electrodes separated by interfinger gaps of 1–10 μm. Species regenerated at the collector electrodes can diffuse back to the generator electrodes for subsequent reduction, thereby amplifying the generator current through redox cycling. This configuration offers high sensitivity with a peroxide low detection limit of 2 fmol, and its reliance on diffusion rather than convection ensures that signals are not affected by rotation-induced disturbances. However, IDAs exhibit an interfacial capacitance that can lead to high impedance. In addition, fabrication via photolithography is both expensive and time-consuming, and the resulting electrode patterns are fragile, making IDAs typically single-use devices, resulting in poor experimental reproducibility. Deposition of technical catalysts on the IDA is not possible, so these systems can only operate with metals that can be directly deposited on the IDA, for instance by electrodeposition.

#### 5.2.2 Full-Cell Setup

The MEA is the most representative configuration for a real PEM system operation. In a full-cell setup, the MEA comprises both cathode and anode electrodes, gas diffusion layers, and a proton-conducting membrane, with the membrane sandwiched between the electrodes. Relatively few attempts have been made to measure hydrogen peroxide within active systems, although Liu and Zuckerbrod used platinum wire microelectrodes to measure hydrogen peroxide concentration within PEM fuel cells, determining that concentration was dependent on the thickness of the Nafion membrane and varied between 5 and 25 ppm under appropriate operating conditions.^18^ More recently, the approach of using such microelectrodes as in situ probes for hydrogen peroxide detection has been expanded.^10,125,126^ More common than the direct measurement of hydrogen peroxide is the use of protocols to emulate situations under which degradation is enhanced. Accelerated stress tests (ASTs) are critical to measure indicators of durability in realistic timeframes. Commonly employed chemical stress test for PEMFC systems include operation at open-circuit voltage (OCV) at 90 °C and 30% relative humidity (RH), or cyclic OCV, in which the membrane is alternately exposed to wet and dry conditions to accelerate oxidative degradation. In electrode-only ASTs, the system is exposed to one or more of the following: elevated temperatures, higher voltages, loading-cyclic (LC) conditions, or start–stop (ST/ST) cycles.^127,128^ The primary goal of ASTs is to accelerate the aging process of the materials used in PEMFCs and PEMWEs to identify potential failure modes and degradation pathways. Fenton’s test is used to assess the durability of components to radical attack, focusing on the durability against radical attack rather than measuring peroxide formation. This method emphasizes mitigating the impact of the attack rather than slowing the rate at which the attack occurs. However, catalyst-coated membrane (CCM) fabrication is time-consuming and requires substantial material quantities, which may be impractical for catalyst developers synthesizing limited batches. Additionally, contamination from airborne impurities and degradation of cell components can affect the test reliability.

## 6 State-of-the-Art H_2_O_2_ Suppression Strategies

As crossover and peroxide/ROS formation are critical degradation factors in PEM technologies, developing suppression strategies is essential to enhancing the efficiency, stability, and safety of PEMWEs and PEMFCs. This section summarizes recent advances in such strategies, focusing on three main stages of intervention, as shown in Figure 16. First, peroxide formation can be prevented by (1) limiting gas crossover and (2) employing catalysts that do not produce peroxide. If some peroxide is produced, then (3) peroxide-to-water decomposition catalysts are used. Second, formation of ROS can be decreased by (4) discouraging peroxide-to-radical formation through reduction in the quantity of Fenton reaction catalysts. For any ROS that still gets produced and to mitigate the damage, (5) radical scavengers or quenchers are introduced. Last is to (6) make materials more resilient toward ROS attack. Key approaches include catalyst engineering, membrane modification, and optimization of system operating conditions. These different aspects are discussed below.

**Figure 16:**
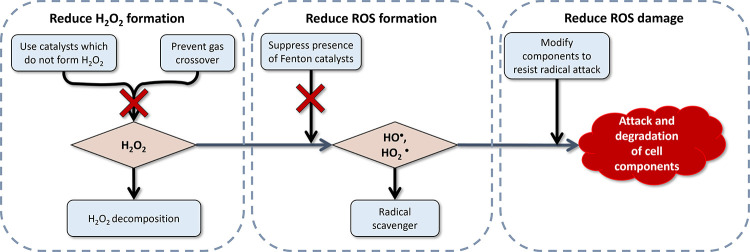
Routes to suppress peroxide/ROS damage.

### 6.1 Prevention of Hydrogen Peroxide Formation

#### 6.1.1 Catalyst Modification

To prevent the formation of peroxide, catalyst modification encompasses surface optimization, active site engineering, surface functionalization, and electrocatalyst support modification. Surface optimization involves tuning the size,^129,130^ the shape,^131^ and the surface facet and nominal loading^132^ of the catalyst nanoparticles to enhance the kinetics of the desired ORR and OER while minimizing peroxide generation. A critical aspect of achieving 4-electron (4e^–^) ORR selectivity involves dissociating the O–O bond in the adsorbed hydroperoxide (HO_2,ad_) species.

Active site engineering includes doping the catalyst with specific elements or incorporating heteroatoms into the catalyst structure^22,133^ to increase selectivity toward the desired reaction. Transitioning from Pt nanoparticles to Pt single-atom catalysts (SACs) appears to be advantageous, as O–O bonds prefer to elongate at the Pt SAC surface, therefore making the HO_2,ad_ destabilized and cleavage of O–O bond favored, thus favoring the 4e^–^ ORR.^134,135^

Carbon black, prone to corrosion and contributing to peroxide formation, can be modified to minimize peroxide generation. This modification can be achieved by chemically or physically altering carbon black itself or replacing it with alternatives. Current work also emphasizes carbon-free or transition metal support materials like doped tin oxide (SnO_2_), molybdenum carbide (Mo_2_C), and titanium carbide (TiC) due to the possibility of superior durability and activity.^136^ However, using transition metal-based supports may exacerbate production of radicals through the Fenton reaction—for instance, SnO_2_ tends to favor the 2-electron pathway, and in fact, Sb-SnO_2_ and other doped oxides are sometimes used deliberately for selective peroxide formation. Dissolution of TiC supports will produce soluble titanium, which is a potent Fenton catalyst (see above). Hence, a holistic approach must be taken when considering improved materials, as fixing one problem (reduced hydrogen peroxide production) may make another problem worse (H_2_O_2_ conversion to ROS).

Modification of catalysts so that they are less likely to produce hydrogen peroxide in the presence of crossover gases is another potential approach. For instance, Shi et al.^121^ developed a Pt-Co alloy HOR catalyst with a stabilized thin Pt skin (one to two atomic layers) supported on high-surface-area carbon black for PEMFCs, which reduced H_ad_ formation on the Pt surface and suppressed peroxide generation due to oxygen crossover (eq 23) in PEMFCs by 50%.

#### 6.1.2 Gas Crossover Suppression and Peroxide Decomposition

To mitigate the gas crossover and decompose any peroxide formed, as mentioned previously, thicker membranes can be used, but this comes at the cost of a greater *iR* drop and increased material cost. Another approach is to insert a proton–conductive gas diffusion barrier within the membrane to act as a physical barrier to reduce crossover. For example, a graphene-based barrier layer has been proposed as the gas-blocking interlayer within the membrane. Mechanistically, protons traverse the graphene framework, whereas larger ions and molecules are sterically hindered and thus barred from these nanochannels. The blocking performance is governed by the interlayer spacing of the graphene-flake network and the lateral flake size, which together set the effective cutoff and tortuosity. Recent studies embedding single-layer graphene on the PEM systems delivered more than 50% lower hydrogen crossover while maintaining operational functionality.^137,138^

However, the most common approach in PEMWEs is the incorporation of a recombination catalyst within the MEA. Although the main purpose of the recombination catalyst is to prevent crossover of oxygen into the hydrogen gas stream and hydrogen into the oxygen gas stream, they also potentially have the subsidiary effect of reducing hydrogen peroxide formation in the catalyst layer due to the crossover of gases. Common catalyst types are summarized in Figure 17.^139^ Mechanistically, using Pt (the most common recombination catalyst) as an example, the peroxide decomposition reaction (Table 2, eq 13) first involves the rate-limiting step (eq 37), where the Pt surface is oxidized to Pt­(O) and then reduced back to peroxide by a second peroxide (eq 38),^140^ as shown below:

37 $$\mathrm{Pt}+H_{2}O_{2\left(\mathrm{aq}\right)}⇄H_{2}O_{\left(l\right)}+\mathrm{Pt}\left(O\right)$$

38 $$\mathrm{Pt}\left(O\right)+H_{2}O_{2\left(\mathrm{aq}\right)}⇄\mathrm{Pt}+O_{2\left(g\right)}+H_{2}O_{\left(l\right)}$$

**Figure 17:**
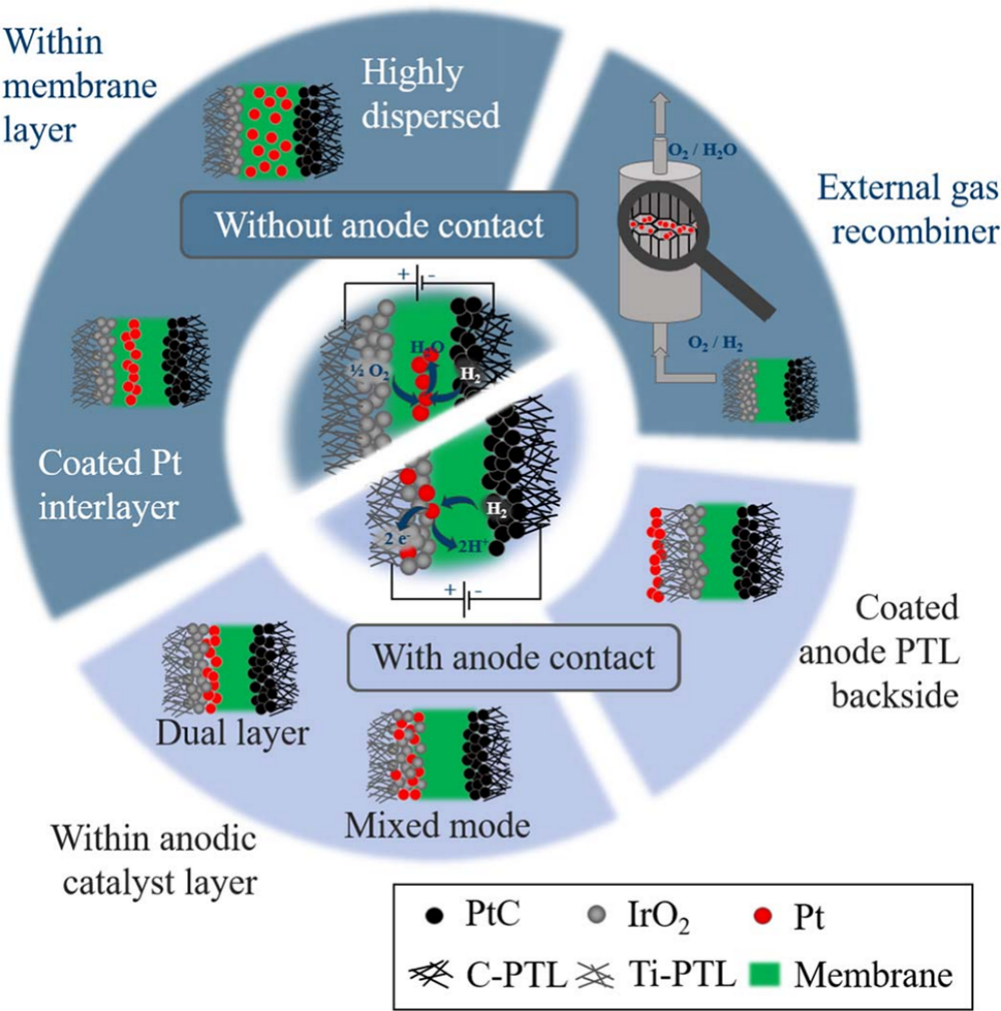
Current strategies to deal with crossover, including addition of a recombination catalyst within a PEMWE to reduce the hydrogen content in the oxygen content on the anode side. MEA strategies such as the implementation of a recombination catalyst within the MEA, on the anode porous transport layer (PTL), within the anodic catalyst layer, or built into the membrane as a thin layer or in a highly dispersed manner reduce the likelihood of hydrogen peroxide generation, whereas external processes such as the use of external gas recombiners will not. Reprinted from ref 139. Copyright 2022 Stähler et al. under the CC-BY 4.0 license.

A comprehensive overview of recombination catalyst insertion position versus durability was given by Fahr et al.^76^ When the recombination catalyst is incorporated into the membrane, the catalyst can also suppress the crossover of oxygen to the hydrogen side (and hydrogen to the oxygen side). However, modification of the ion exchange capacity (IEC) by the introduced recombination catalyst is a vital factor researchers should consider when developing membrane modification. Though recombination can reduce the ROS, introducing a recombination catalyst can inadvertently reduce the number of ionizable sites, which affects the proton conductivity. A higher IEC provides more proton-conducting sites, promoting the complete reduction of oxygen to water and minimizing the partial reduction of hydrogen peroxide. Recently, Xie et al.^141^ developed a PFSA-anchoring approach that disperses Pt nanoparticles within hydrophilic, H^+^/ H_2_-permeable channels, simultaneously hindering H_2_ diffusion and providing recombination activity. This distribution reduced the H_2_-in-O_2_ level below the LFL while maintaining membrane durability for more than 500 h using a 60 μm thin membrane.

### 6.2 Prevention of ROS Formation

#### 6.2.1 Controlling the Presence of Fenton Catalysts

To avoid ROS generation, removal of impurities from the system is essential, as these can drive Fenton reactions. Impurities may be introduced by failure of water purification (Cl^–^), external contamination (organics, e.g., leached from polymers), Cu^2+^ or (stainless) steel tubing degradation (Fe^2+^, Fe^3+^), etc.^74^ To minimize impurities, pretreating the membrane with H_2_SO_4_ acid has been shown to be effective,^142^ as has the use of Fe-free end plates.^143,144^ In PEMWEs, the use of a platinized Ti separator demonstrated a 10-fold reduction in degradation rate, presumably due to a reduction in soluble titanium species.^145^

#### 6.2.2 Radical Quenching

To removing existing ROS, transition metals^146,147^ such as cerium and manganese function as effective radical quenchers due to their self-regeneration capabilities. The radical quenching processes are in some ways the mirror of the Fenton process in that they involve the cycling of a metal ion (or other redox species) between two oxidation states, Table 5.^16^ However, compared to catalysts for the Fenton reaction, the redox transition needs to occur at a relatively high potential (*E*_M^*n*^/M^*n*+1^_^o^ = 1.504 and 1.72 V for Mn^2+^ and Ce^3+^, respectively).

**Table 5:** Summary of Radical Quenching Reactions with Reaction Rate and Free Energy of Reaction for Ce^3+^ and Mn^2+^ as Examples^148,149^

		Rate constant (M^–1^ s^–1^)		**Δ_r_ *G*° (kJ mol^–1^)^a^**	
**Reaction**		**Ce^3+^**	**Mn^2+^**	**Ce^3+^**	**Mn^2+^**
M_(aq)_^*n*+^ + HO_(aq)_^•^ + H_(aq)_^+^ → M_(aq)_^(*n*+1)+^ + H_2_O_(l)_	(39)	3 × 10^8^	3 × 10^7^	–97	–118
M_(aq)_^(*n*+1)+^ + H_2_O_2(aq)_ → M_(aq)_^*n*+^ + HO_2 (aq)_^•^ + H_(aq)_^+^	(40)	10^6^	3 × 10^3^	–25	–4
M_(aq)_^(*n*+1)+^ + HO_2 (aq)_^•^ → M_(aq)_^*n*+^ + O_2(g)_ + H_(aq)_^+^	(41)	2.7 × 10^6^	<10^5^	–173	–152

a These values have been calculated using the standard free energy of formation of the respective species provided in the Supporting Information (Table S4).

However, conventional radical quenchers in the membrane tend to migrate within the membrane and interact with ionomeric chains, which will affect their efficacy. Xu et al.^150^ developed a non-destructive swelling–filling method to incorporate ceria into the Nafion membrane as a radical scavenger. Recently, radical quenchers incorporated into a metal–organic framework (MOF) were developed^151^ to immobilize Ce within the structure. The introduction of the MOF significantly improved the IEC, ionic conductivity, and stability of the Ce-containing membrane. Complex materials, such as multicore–shell structured Pt@CeO_2_, leverage the strong metal–support interaction (SMSI) effect,^152^ achieving only 7% the lowest fluoride ion emission rate found in the cerium-free benchmark systems during the Fenton test. Peroxide decomposition catalysts, such as zirconia nanoparticles (ZrO_2_)^148,149^ modified and co-doped with CeO_2_ on Pt, have been developed to provide bifunctional activity—both recombining crossover gases and scavenging radical scavengers.^153^

### 6.3 Membrane Engineering against ROS Attack

The very beginning of the PEM approach for fuel cells used copolymers of sulfonated polystyrene and divinylbenzene as membranes which were short-lived, with 50 wt% loss in 8 h in accelerated chemical stability (Fenton) tests. DuPont’s Nafion (PFSA) was introduced in the late 1960s and rapidly displaced them due to its far superior chemical stability (0.5 wt% loss in 8 h under equivalent conditions). Recent interest has shifted to sulfonated hydrocarbon membranes again, like sulfonated poly-ether-ether-ketone (sPEEK),^154^ sulfonated polysulfone (sPSU),^155^ sulfonated polyimides (sPIs),^156^ and polybenzimidazoles (PBIs).^157^ These sulfonated hydrocarbon membranes offers cost and performance benefits but typically demand antioxidant/additive strategies to manage their greater susceptibility to ROS-induced degradation relative to PFSA under comparable stress tests.^158^ Recent studies have explored non-PFSA membranes to both reduce hydrogen crossover and mitigate the PFSA-specific “unzipping” degradation pathway, e.g., fluorine-free MEAs with sulfonated poly­(phenylene sulfone) (sPPS).^159^ These employ an aromatic hydrocarbon backbone with sulfone linkages and no perfluoroether side chains or PFSA-type end groups; such an engineering design significantly eliminates the membrane unzipping route. However, ROS-driven degradation via other pathways (e.g., aromatic ring attack, desulfonation, and sulfone-link scission) can still occur.^160^ Another approach is to incorporate redox-active moieties that intercept ROS, such as immobilized ceria or phosphonic acid functionalities, which demonstrably lower fluoride emission rates under Fenton/OCV tests.^161^

## 7 Summary and Future Outlook

This review examines hydrogen peroxide formation and its consequences in PEM fuel cells and PEM water electrolyzers, highlighting gas crossover as a major contributor alongside catalyst-driven electrochemical pathways. We provide a comprehensive, fundamental overview of the thermodynamics and mechanisms of peroxide and ROS formation from both chemical and electrochemical perspectives. We also discuss gas crossover from an operational standpoint, noting that it is promoted by high membrane gas permeability, elevated relative humidity, high temperature, large pressure gradients, and electro-osmotic drag. The resulting PEM system failure modes are summarized, including (1) hot spot formation, (2) membrane “unzipping”, (3) catalyst redeposition and subsequent band formation, and (4) oxidation of carbon-based components, all of which can impair conductivity, water uptake, and durability.

This review further compares peroxide/ROS detection strategies. Most peroxide formation studies rely on half-cell RRDE measurements, which primarily probe electrochemical pathways, while crossover-driven and other chemical formation pathways are typically investigated in full cells. However, the complexity of full-cell operation can hinder the mechanistic interpretation of peroxide/ROS formation. Despite these challenges, advances in in situ techniques now allow peroxide/ROS formation to be probed under realistic full-cell conditions. Finally, we summarize progress in peroxide suppression strategies, including catalyst modification, membrane engineering, and optimization of the operating conditions. These advances show promise for extending PEM systems’ lifetimes and enabling thinner membranes in CCMs. However, the incorporation of recombination catalysts or radical scavengers and the mitigation of membrane cracking must be carefully balanced and mechanistically validated to support the development of efficient, durable, and safe devices.

Looking ahead, to advance the understanding of peroxide and ROS and improve the longevity of PEM systems, future research could focus on the following:

1. Most work to date has focused on hydrogen crossover; thus, greater attention to oxygen crossover is encouraged, as understanding this process is critical for identifying efficiency losses and ultimately improving cell longevity. In particular, oxygen crossover can lead to local mixed potentials that promote peroxide and ROS formation. Decoupling oxygen crossover from the other variables is helpful for understanding the structure–property–performance relationships, which in turn can guide the design of the membrane and catalyst.
2. In parallel, the development of half-cell detection techniques that can more comprehensively elucidate peroxide formation mechanisms remains an important priority. Current half-cell approaches primarily quantify electrochemically generated peroxide under simplified conditions, whereas crossover-driven and chemically mediated pathways are often inferred indirectly in full cells. We encourage the study of half-cell techniques for pathway-resolved analysis, including (but not limited to) distinguishing electrochemical and chemical contributions and capturing transient behavior, while keeping the methods compatible with realistic catalyst layers and ionomer environments. In addition, most in situ detection methods are limited to qualitative mechanistic interpretation, partly due to the short lifetimes of ROS species—there is still a gap in bridging this to robust quantitative assessment.

A number of researchers are pursuing a course of developing electrolyzers that explicitly produce hydrogen peroxide as a product. In these systems, particular care needs to be taken to avoid decomposition of peroxide into ROS and to ensure the suitable degradation of any produced ROS so as not to significantly decrease the operating lifetime of those systems.

## Supplementary Material



## Notes

List of abbreviations; discussion of thermodynamic equivalence of gas, aqueous, and adsorbed species; summary of HER/HOR and their elementary steps; summary of 1-, 2-, and 4-electron ORR and their elementary steps; summary of 1-, 2-, and 4-electron WOR and their elementary steps; standard state Δ_f_G° values for all species used in calculations (PDF)
