# Supplementary material for: From Formation to Failure: The Role of Hydrogen Peroxide in Proton Exchange Membrane Technologies
Source: ACS Catal. 2026 Feb 16;16(5):4266–89. doi: 10.1021/acscatal.5c08411 (PMC12973266; doi:10.1021/acscatal.5c08411)
Supplement: Supplementary file 1 [file cs5c08411_si_001.pdf]

Supplementary Information

From Formation to Failure: The Role of Hydrogen Peroxide in PEM Technologies

Tingting Mo[a], Christopher M. Zalitis[b], Colleen Jackson[b], Enrico Petrucco[b], Jonathan Sharman[b], Anthony R.J. Kucernak*[a]

a. Imperial College London, Department of Chemistry, Molecular Science Research Hub, London, W12 0BZ, UK

b. Johnson Matthey Technology Centre, Blounts Court Road, Sonning Common, Reading, RG4 9NH, UK

*Corresponding author: anthony@imperial.ac.uk

LIST OF ABBREVIATIONS

ABTS	2,2'-Azino-Bis(3-Ethylbenzothiazoline-6-Sulfonate)	*ORR*	Oxygen Reduction Reaction
AEM	Adsorbate Evolution Mechanism	*PBI*	Polybenzimidazole
AST	Accelerated Stress Tests	*PCET*	Proton Coupled Electron Transfer
CCM	Catalyst-Coated Membrane	*PEM*	Proton-Exchange Membrane
DDECFC	Double-Disk-Electrode Channel Flow Cell	*PEMFC*	Proton Exchange Membrane Fuel Cells
ESR	Electron Spin Resonance	*PEMWE*	Proton Exchange Membrane Water Electrolyzers
FER	Fluorine Emission Rate	*PFSA*	Perfluoro Sulfonic Acid
GAME	Gas-Accessible Membrane Electrode	*PTFE*	Polytetrafluoroethylene
GDL	Gas Diffusion Layer	*RH*	Relative Humidity
HER	Hydrogen Evolution Reaction	*ROS*	Reactive Oxygen Species
HOR	Hydrogen Oxidation Reaction	*RRDE*	Rotating Ring-Disk Electrode
HRP	Horseradish Peroxidase	*SAC*	Single-Atom Catalysts
IDA	Interdigitated Array Electrode	*SECM*	Scanning Electrochemical Microscopy
IEC	Ion Exchange Capacity	*SMSI*	Strong Metal-Support Interaction
LC	Loading-Cyclic	*sPEEK*	Sulfonated Poly-Ether-Ketone-Ether
LFL	Lower Flammability Limit	*sPI*	Sulfonated Polyimide
LOM	Lattice Oxygen Participation Mechanism	*sPPS*	Sulfonated Poly Phenylene Sulfone
MEA	Membrane Electrode Assembly	*sPSU*	Sulfonated Polysulfone
MOF	Metal–Organic Framework	*ST/ST*	Start-Stop
MPL	Microporous Layers	*UME*	Ultramicroelectrode
OCV	Open-Circuit Voltage	*WOR*	Water Oxidation Reaction
OER	Oxygen Evolution Reaction		

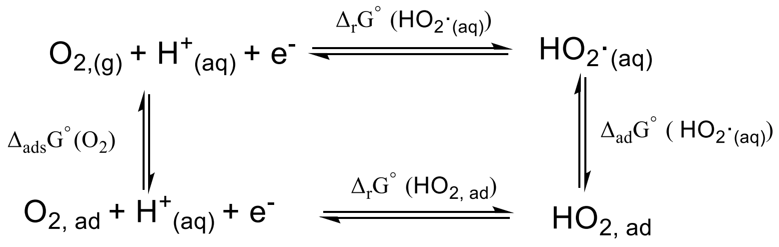

Figure S 1 Illustration of thermodynamic equivalence between species. $O_{2(g)}$ represents the standard state of O_2 which is assumed to be in equilibrium with dissolved oxygen $O_{2(aq)}$. The free aqueous species require an adsorption energy ($\Delta_{ad}G^\circ$) to adsorb on the surface

It is important to distinguish between the free species in aqueous solution and the adsorbed species, as there are differences in the free energy of adsorption for oxygen containing species onto catalytic surfaces. The electrochemical potential for forming the adsorbed species differs from that of the solvated species, associated with energy of adsorption of the product and reactant, as shown in Figure S 1. Therefore, while the solution phase reaction gives the overall thermodynamics of the reaction, the adsorption/desorption energy should also be considered in the net reaction. It is important to take extra care when discussing adsorbed reactions compared to solution phase reactions, as computational estimates of adsorption energies may not suitably take into account the appropriate electrochemical environment seen by the adsorbate (solvent, spectator ions etc).

For comparison with literature values, the gaseous species are assumed to be in equilibrium with the solution at 1 bar pressure (i.e.$O_{2(g)} \rightleftharpoons O_{2(aq)}$, $H_{2(g)} \rightleftharpoons H_{2(aq)}$; $\Delta_r G$ = 0.00 J mol^{-1}), and soluble species (aq) are assumed to be present at m = 1 mol kg^{-1}. For the sake of simplicity, we ignore some possible species (singlet oxygen, ozone) and also limit the number of interconversion reactions as our purpose is to consider those conditions under which reactive oxygen species such as hydrogen peroxide may be produced.

*Table S 1 Summary of elementary steps in Hydrogen Oxidation/Hydrogen Evolution Reactions and the overall reaction of HOR/HER. The * represents the free site on the surface of the metal catalyst, H_{ad} represents the adsorbed hydrogen atom, $H_{2, (aq)}$ represents the dissolved hydrogen gaseous species which is in equilibrium with the hydrogen gas.*

Reaction type	Reaction	
Hydrogen Oxidation Reaction	$\boldsymbol{H_{2(g)} \longrightarrow 2H^+_{(aq)} + 2e^-}$	**(1)**
Hydrogen Evolution Reaction	$\boldsymbol{2H^+_{(aq)} + 2e^- \longrightarrow H_{2(g)}}$	**(2)**
Tafel reaction	$2H^\bullet{}_{ad} \rightleftharpoons H_{2(g)} + 2*$	(3)
Heyrovsky reaction	$H^\bullet{}_{ad} + H^+_{(aq)} + e^- \rightleftharpoons H_{2(g)}$	(4)
Volmer reaction	$H^+_{(aq)} + e^- + * \rightleftharpoons H^\bullet{}_{ad}$	(5)

*Table S 2 Summary of Oxygen Reduction Reaction (ORR) mechanisms. The * represents the free site on the surface of the catalyst, OOH_{ad}, OH_{ad}, O_{ad} represent the adsorbed species on the catalyst surface.*

Reaction type	Reaction	
Associative mechanism: OOH_{ad}, OH_{ad}, O_{ad}		
1 e^- ORR ($E^o = 0.152$ V vs RHE)	$\boldsymbol{O_{2(g)} + H^+_{(aq)} + e^- \rightleftarrows HO^{\bullet}_{2(aq)}}$	**(6)**
Outer sphere reaction ($E^o = -0.137$ V vs RHE)[1]	$O_{2(g)} + e^- \rightleftarrows O^{\bullet-}_{2\,(aq)}$	(7)
	$O^{\bullet-}_{2,(aq)} + H^+_{(aq)} \rightleftarrows HO^{\bullet}_{2(aq)}$	(8)
Interfacial reaction	$O_{2(g)} + H^+_{(aq)} + e^- \rightleftarrows OOH_{ad}$	(9)
	$HO_{2,ad} \rightleftarrows HO^{\bullet}_{2\ (aq)} + *$	(10)
2 e^- ORR ($E^o = 0.695\ V\ vs\ RHE$)	$\boldsymbol{O_{2(g)} + 2H^+_{(aq)} + 2e^- \rightleftarrows H_2O_{2(aq)}}$	**(7)**
	$O_{2(g)} + H^+_{(aq)} + e^- + * \rightleftarrows HO_{2,ad}$	(9)
	$HO_{2,ad} + H^+_{(aq)} + e^- \rightleftarrows H_2O_{2(aq)} + *$	(11)
4 e^- ORR ($E^o = 1.229\ V\ vs\ RHE$)	$\boldsymbol{O_{2(g)} + 4H^+_{(aq)} + 4e^- \rightleftarrows 2H_2O_{(l)}}$	**(6)**
	$O_{2(g)} + H^+_{(aq)} + e^- + * \rightleftarrows HO_{2,ad}$	(9)
	$HO_{2,ad} + H^+_{(aq)} + e^- \rightleftarrows O_{ad} + H_2O_{(aq)}$ $or\ (dissociative)\ HO_{2,ad} + * \rightleftarrows OH_{ad} + O_{ad}$	(12)
	$O_{ad} + H^+_{(aq)} + e^- \rightleftarrows OH_{ad}$	(13)
	$OH_{ad} + H^+_{(aq)} + e^- \rightleftarrows H_2O_{(l)} + *$	(14)
Dissociative (2 site) mechanism: OH_{ad}, O_{ad}		
4 e^- ORR ($E^o = 1.229\ V\ vs\ RHE$)	$\boldsymbol{O_{2(g)} + 4H^+_{(aq)} + 4e^- \rightleftarrows 2H_2O_{(aq)}}$	**(15)**
	$1/2\ \ O_{2(g)} \rightleftarrows O_{ad}$	(16)
	$O_{ad} + H^+_{(aq)} + e^- \rightleftarrows OH_{ad}$	(13)
	$OH_{ad} + H^+_{(aq)} + e^- \rightleftarrows H_2O_{(l)} + *$	(14)

*Table S 3 Summary of Water Oxidation Reaction (WOR) mechanisms. The * represents the free site on the surface of the catalyst, $HO_{2\,ad}$, OH_{ad}, O_{ad} represent the adsorbed species on the catalyst surface*[2-5]

Reaction type	Reaction	
1 e⁻ WOR ($E^o = 2.81\text{V}\ vs\ RHE$)	$\boldsymbol{H_2O_{(l)} \rightleftarrows HO^{\bullet}_{(aq)}} + H^+_{(aq)} + \boldsymbol{e^-}$	**(17)**
	$H_2O_{(l)} + * \rightleftarrows OH_{ad} + H^+_{(aq)} + e^-$	(18)
	$OH_{ad} \rightleftarrows HO^{\bullet}_{(aq)} + *$	(19)
2 e⁻ WOR ($E^o = 1.77\ V\ vs\ RHE$)	$\boldsymbol{2H_2O_{(l)} \rightleftarrows H_2O_{2(aq)} + \ 2H^+_{(aq)} + 2e^-}$	**(20)**
	$H_2O_{(l)} + * \rightleftarrows OH_{ad} + H^+_{(aq)} + e^-$	(18)
	$OH_{ad} + H_2O_{(l)} \rightleftarrows H_2O_{2(aq)} + * + H^+_{(aq)} + e^-$	(21)
4 e⁻ WOR ($E^o = 1.229\ V\ vs\ RHE$)	$\boldsymbol{2H_2O_{(l)} \rightleftarrows O_{2(g)} + 4H^+_{(aq)} + 4e^-}$	**(20)**
	$H_2O_{(aq)} + * \rightleftarrows OH_{ad} + H^+_{(aq)} + e^-$	(18)
	$OH_{ad} \rightleftarrows O_{ad} + H^+_{(aq)} + e^-$	(22)
	$O_{ad} + H_2O_{(aq)} \rightleftarrows \ HO_{2,ad} + H^+_{(aq)} + e^-$	(23)
	$HO_{2,ad} \rightleftarrows O_{2(g)} + H^+_{(aq)} + e^- + *$	(24)

Table S4 Free energy of formation under standard conditions (298.15K, 1 bar) for species used in calculated reaction free energies, electrochemical potential, and pK_a's in Table 2, Table 3, Figure 4 and Table 5.

Material	State	$\Delta_f G^o$/kJ mol^{-1}	±error /kJ mol^{-1}	Reference
O_2	g	0		standard state
H_2	g	0		standard state
H^+	aq	0		standard state
$H\cdot$	aq	223	2	6
$O_2{\cdot}^-$	aq	33.8	1.9	6
$HO_2\cdot$	aq	7	2	6
$O{\cdot}^-$	aq	93.1	1.7	6
$HO\cdot$	aq	26.3	1.6	6
H_2O_2	aq	-134.1		7
HO_2^-	aq	-67.4		7
H_2O	aq	-237.178		7
OH^-	aq	-157.293		7
Fe^{2+}	aq	-91.2		7
Fe^{3+}	aq	-16.8		7
Mn^{2+}	aq	-228.1		7
Mn^{3+}	aq	-83		7
Ce^{3+}	aq	-676		7
Ce^{4+}	aq	-510		7

Reaction free energies, electrochemical potentials, and pK_a's in Table 2, Table 3, and Figure 4 were calculated under standard conditions using the Free Energies of formation of the respective species provided in Table S4 using the Hess cycle approach. These free energies of formation were obtained from appropriate sources[6, 7] and carefully checked. Furthermore, the resultant reaction free energies/electrochemical potentials/pKa's were checked for internal consistency. The redox couples for each of the metals mentioned: Fe^{2+}/Fe^{3+},Mn^{2+}/Mn^{3+},Ce^{3+}/Ce^{4+}, are calculated using the above data to be $E^o_{Fe2+/Fe3+}$=0.771 V; $E^o_{Mn2+/Mn3+}$ =1.504 V; $E^o_{Ce3+/Ce4+}$=1.720 V, respectively, which accords well with the tabulated values[7].

Reference

(1) Petlicki, J.; van de Ven, T. G. M. The equilibrium between the oxidation of hydrogen peroxide by oxygen and the dismutation of peroxyl or superoxide radicals in aqueous solutions in contact with oxygen. *J Chem Soc Faraday T* **1998**, *94* (18), 2763-2767. DOI: DOI 10.1039/a804551h.

(2) Shi, X.; Back, S.; Gill, T. M.; Siahrostami, S.; Zheng, X. Electrochemical Synthesis of H2O2 by Two-Electron Water Oxidation Reaction. *Chem* **2021**, *7* (1), 38-63. DOI: 10.1016/j.chempr.2020.09.013.

(3) Zhang, Y. Y.; Fu, Q.; Song, B.; Xu, P. Regulation Strategy of Transition Metal Oxide-Based Electrocatalysts for Enhanced Oxygen Evolution Reaction. *Accounts of Materials Research* **2022**, *3* (10), 1088-1100. DOI: 10.1021/accountsmr.2c00161.

(4) Chen, F. Y.; Wu, Z. Y.; Adler, Z.; Wang, H. T. Stability challenges of electrocatalytic oxygen evolution reaction: From mechanistic understanding to reactor design. *Joule* **2021**, *5* (7), 1704-1731. DOI: 10.1016/j.joule.2021.05.005.

(5) Kuznetsova, E.; Cuesta, A.; Thomassen, M. S.; Sunde, S. Identification of the byproducts of the oxygen evolution reaction on Rutile-type oxides under dynamic conditions. *Journal of Electroanalytical Chemistry* **2014**, *728*, 102-111. DOI: 10.1016/j.jelechem.2014.06.031.

(6) Armstrong, D. A.; Huie, R. E.; Koppenol, W. H.; Lymar, S. V.; Merényi, G.; Neta, P.; Ruscic, B.; Stanbury, D. M.; Steenken, S.; Wardman, P. Standard electrode potentials involving radicals in aqueous solution: inorganic radicals (IUPAC Technical Report). *Pure and Applied Chemistry* **2015**, *87* (11-12), 1139-1150. DOI: doi:10.1515/pac-2014-0502 (acccessed 2025-12-16).

(7) Bard, A. J.; Parsons, R.; Jordan, J. *Standard Potentials in Aqueous Solution*; Routledge, 2017. DOI: 10.1201/9780203738764.
